# Startle responses in Duchenne muscular dystrophy: a novel biomarker of brain dystrophin deficiency

**DOI:** 10.1093/brain/awac048

**Published:** 2022-02-08

**Authors:** Kate Maresh, Andriani Papageorgiou, Deborah Ridout, Neil A Harrison, William Mandy, David Skuse, Francesco Muntoni

**Affiliations:** Dubowitz Neuromuscular Centre, UCL Great Ormond Street Institute of Child Health, London WC1N 1EH, UK; Queen Square Centre for Neuromuscular Diseases, University College London, London WC1N 3BG, UK; Dubowitz Neuromuscular Centre, UCL Great Ormond Street Institute of Child Health, London WC1N 1EH, UK; Population, Policy and Practice Research and Teaching Department, UCL Great Ormond Street Institute of Child Health, London WC1N 1EH, UK; NIHR Great Ormond Street Hospital Biomedical Research Centre, UCL Great Ormond Street Institute of Child Health, London WC1N 1EH, UK; Division of Psychological Medicine and Clinical Neurosciences, School of Medicine, Cardiff University, Cardiff CF14 4XN, UK; Department of Clinical, Educational and Health Psychology, University College London, London WC1E 6BT, UK; Department of Behavioural and Brain Sciences, UCL Great Ormond Street Institute of Child Health, London WC1N 1EH, UK; Dubowitz Neuromuscular Centre, UCL Great Ormond Street Institute of Child Health, London WC1N 1EH, UK; Queen Square Centre for Neuromuscular Diseases, University College London, London WC1N 3BG, UK; NIHR Great Ormond Street Hospital Biomedical Research Centre, UCL Great Ormond Street Institute of Child Health, London WC1N 1EH, UK

**Keywords:** Duchenne muscular dystrophy, dystrophin, brain, fear conditioning, anxiety

## Abstract

Duchenne muscular dystrophy (DMD) is characterized by loss of dystrophin in muscle, however patients also have variable degree of intellectual disability and neurobehavioural co-morbidities. In contrast to muscle, in which a single full-length dystrophin isoform (Dp427) is produced, multiple isoforms are produced in the brain, and their deficiency accounts for the variability of CNS manifestations, with increased risk of comorbidities in patients carrying mutations affecting the 3′ end of the gene, which disrupt expression of shorter Dp140 and Dp71 isoforms. A mouse model (*mdx* mouse) lacks Dp427 in muscle and CNS and exhibits exaggerated startle responses to threat, linked to the deficiency of dystrophin in limbic structures such as the amygdala, which normalize with postnatal brain dystrophin-restoration therapies. A pathological startle response is not a recognized feature of DMD, and its characterization has implications for improved clinical management and translational research.

To investigate startle responses in DMD, we used a novel fear-conditioning task in an observational study of 56 males aged 7–12 years (31 affected boys, mean age 9.7 ± 1.8 years; 25 controls, mean age 9.6 ± 1.4 years). Trials of two neutral visual stimuli were presented to participants: one ‘safe’ cue presented alone; one ‘threat’ cue paired with an aversive noise to enable conditioning of physiological startle responses (skin conductance response and heart rate). Retention of conditioned physiological responses was subsequently tested by presenting both cues without the aversive noise in an ‘Extinction’ phase. Primary outcomes were the initial unconditioned skin conductance and change in heart rate responses to the aversive ‘threat’ and acquisition and retention of conditioned responses after conditioning. Secondary and exploratory outcomes were neuropsychological measures and genotype associations.

The mean unconditioned skin conductance response was greater in the DMD group than controls [mean difference 3.0 µS (1.0, 5.1); *P* = 0.004], associated with a significant threat-induced bradycardia only in the patient group [mean difference –8.7 bpm (–16.9, –0.51); *P* = 0.04]. Participants with DMD found the task more aversive than controls, with increased early termination rates during the Extinction phase (26% of DMD group versus 0% of controls; *P* = 0.007).

This study provides the first evidence that boys with DMD show similar increased unconditioned startle responses to threat to the *mdx* mouse, which in the mouse respond to brain dystrophin restoration. Our study provides new insights into the neurobiology underlying the complex neuropsychiatric co-morbidities in DMD and defines an objective measure of this CNS phenotype, which will be valuable for future CNS-targeted dystrophin-restoration studies.

## Teaser

Maresh *et al.* report that boys with Duchenne muscular dystrophy (DMD) have greater startle responses to threat than controls, similar to the responses seen in a DMD mouse model that normalise with dystrophin restoration. These responses may be useful outcomes in future clinical trials of CNS dystrophin restoration in DMD.

## Introduction

**Figure 1:**
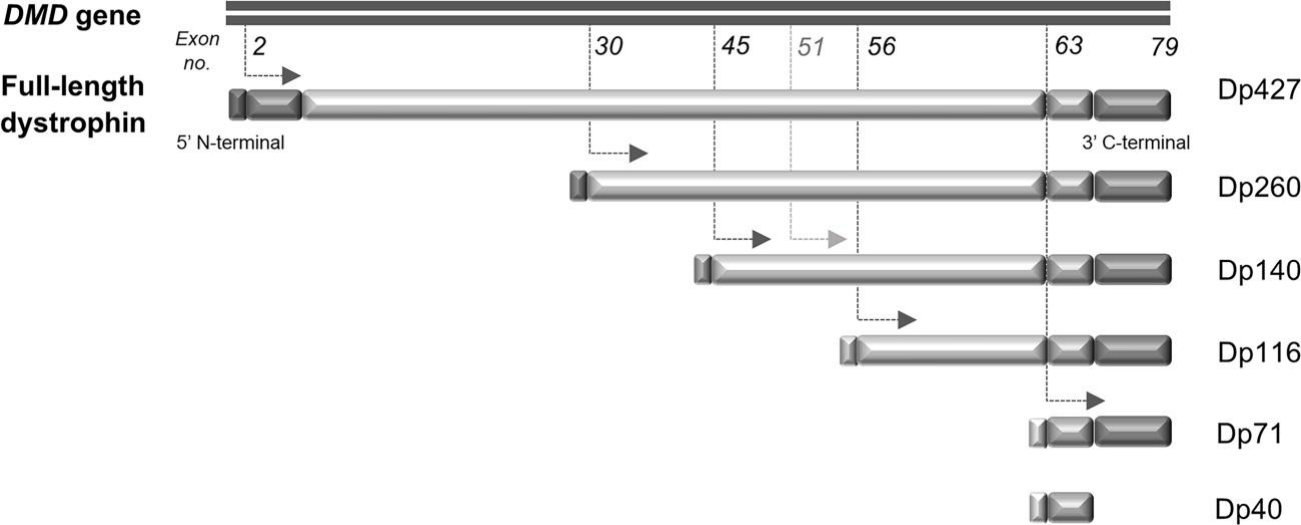
**Schematic representation of *DMD* genomic organization.** The *DMD* gene, which encodes the protein dystrophin, is located on Xp21.2 and comprises 79 exons and seven promoters linked to unique first exons. Dotted arrows indicate the splice sites of these different internal promoters, which splice into the indicated exons (or preceding introns in the case of Dp140) to generate multiple dystrophin isoforms, shown in succession below the full-length dystrophin protein. The isoforms are named by their size in kDa. The three 427 kDa isoforms are Dp427m (muscle), Dp427c (cerebral) and Dp427p (Purkinje); shorter isoforms are Dp260, Dp140, Dp116 and Dp71, the latter of which can be further alternatively spliced to form a 40 kDa Dp40 isoform. Each isoform has a different 5′ N-terminal domain, and all retain the same 3′ C-terminal domain. Numbers in italics indicate the exon number. In the case of the Dp140 isoform, transcription starts at intron 44 (just upstream of exon 45) but translation of the protein does not start until exon 51 (light grey arrow), leading to a long UTR from intron 44–exon 50. It is difficult to predict the effect on Dp140 expression of mutations in this untranslated region. Adapted from figure in Muntoni *et al*.^2^

Duchenne muscular dystrophy (DMD) is an X-linked, life-limiting, progressive neuromuscular disorder with onset in early childhood, caused by mutations in the *DMD* gene encoding the protein dystrophin.^1^ The *DMD* gene is large (2.2 Mb) and comprises 79 exons. It contains seven promoters for different dystrophin isoforms whose expression starts at different exons in the gene: three full-length 427 kD isoforms (muscle, Dp427m; cerebral, Dp427c; Purkinje Dp427p, where 427 represents the molecular weight of the protein) and five shorter isoforms (Dp260, Dp140, Dp116 and Dp71, with its splice variant isoform Dp40), that exhibit developmental, regional and cell-type specificity within different tissues (Fig. 1).^2^ In the human brain, the full-length isoforms Dp427m and Dp427c are present throughout the cortex and basal ganglia with the highest expression in the hippocampus and amygdala and lowest in the cerebellum, whereas Dp427p is expressed at lower levels compared to the remaining full length isoforms.^3,4^ The shorter Dp140, Dp71 and Dp40 isoforms are also expressed in the brain, whilst Dp260 and Dp116 isoforms are not.^3^ Dp140 is expressed at higher levels during development, with lower expression throughout adulthood, and Dp71 and Dp40 are alternative spliced products of the same promoter, with Dp71 being ubiquitously expressed.^3,5^

In all people with DMD, loss-of-function *DMD* gene mutations cause deficiency of full-length Dp427 dystrophin, leading to progressive skeletal muscle and cardiac dysfunction. The location of the mutation determines whether expression of shorter dystrophin isoforms is preserved or lost (Fig. 1). Mutations upstream of exon 45 (towards the 5′ end of the gene) do not affect expression of the Dp140 isoform, whilst those downstream of exon 50 cause loss of Dp140 expression. Mutations within exons 45–50 have an indeterminate effect on Dp140 expression, due to a long untranslated region (UTR) encompassing these exons. Distal *DMD* mutations, from exon 63 to the 3′ C-terminal end, affect expression of the Dp71 isoform as well as Dp140.

Approximately half of individuals with DMD are affected by a complex neuropsychiatric phenotype, which can include intellectual disability, language delay, autism spectrum disorder (ASD), attention-deficit-hyperactivity disorder (ADHD), emotional disorders and obsessive-compulsive disorder.^6,7^ Intellectual disability is strongly related to mutations disrupting expression of Dp140 and Dp71 isoforms. Cumulative loss of these isoforms is associated with increased intellectual impairment: boys deficient only in Dp427 have a mean IQ of 96; those also lacking Dp140 have a mean IQ of <75; the mean IQ for those lacking Dp140 and Dp71 is <50.^8–10^ Some previous studies have suggested an increased risk of emotional, behavioural and neurodevelopmental disorders in individuals lacking the shorter isoforms, including an increased risk of internalizing problems (encompassing anxiety and depressive symptoms), but these findings vary across studies.^11–13^ These isoform-related differences, along with the heterogeneous nature of the neuropsychiatric phenotype in DMD, suggest that several pathophysiological mechanisms are involved.

A mouse model of DMD, the *mdx* mouse, lacks full-length Dp427 but retains shorter isoforms due to a nonsense mutation in exon 23 of the *Dmd* gene.^14^ The CNS phenotype of the *mdx* mouse includes impairments in long-term spatial and recognition memory, learning, cognitive flexibility and social communication,^15–18^ as well as increased fearfulness and stress reactivity, such as enhanced defensive ‘freezing’ startle responses during mild behavioural stress^19^ or foot shock.^20^ Recent work comparing the *mdx* mouse with a mouse model lacking both Dp427 and Dp140 (*mdx52*) found no differences in startle responses between the *mdx* and *mdx52* mice, although *mdx52* mice showed increased anxiety behaviours.^21^ The parallels between the CNS phenotype in the *mdx* mouse and humans with DMD suggest that much of this enhanced defensive behaviour is due to loss of full-length Dp427 dystrophin and that Dp140 deficiency may enhance this pathological phenotype.

In wild-type mice, Dp427 dystrophin co-localizes with a subset of GABA_A_-receptors in the cerebral cortex, amygdala, hippocampus and cerebellum.^20^ GABA_A_-receptor distribution is decreased in *mdx* mouse amygdalae and a recent human SPECT study also demonstrated a reduction of GABA_A_-receptors in the prefrontal cortex of adults with DMD.^22^ The Dp140 isoform is closely co-expressed with genes involved in early neurodevelopmental processes in humans.^3^ Murine studies demonstrate the role of Dp71 in glutamatergic transmission and glial-dependent extracellular ion homeostasis, and Dp40 might play a crucial role in presynaptic functions in the brain.^23^

The reduced GABA_A_-receptor density in the hippocampus and amygdala is considered central to the pathological fear responses of the *mdx* mouse.^24,25^ Postnatal cerebral dystrophin restoration in *mdx* mice can be achieved using antisense oligonucleotides (AON), which induce the skipping of the mutation-carrying exon 23 to cause an in-frame deletion that allows a shortened but functional protein to be produced. Several studies have shown that this technique can restore both dystrophin expression in the amygdala and postsynaptic GABA_A_-receptor density and normalize the pathological fear response.^20,26^ Systemic AON therapies that induce exon-‘skipping’ to convert out-of-frame *DMD* mutations into ‘in-frame’ mutations that allow the production of shortened but functional proteins are in current clinical trials or approved therapies in some countries.^27^ These AONs do not cross the blood_­_–brain barrier, although intrathecal administration would potentially allow brain dystrophin restoration.

These findings raise the intriguing possibility that AON therapies directed to restoring expression of brain dystrophin could also address some of the CNS comorbidities in DMD. Given the increasing life-expectancy in DMD with current standards of care and emerging therapies, improving neuropsychiatric symptoms could significantly benefit quality of life. In order for any therapy to be evaluated, outcome measures are needed to evaluate the effectiveness of dystrophin-restoration. The reversibility of the startle response in the *mdx* mouse after dystrophin-restoration therapy led us to propose that a similar phenotype may be present in boys with DMD. However, startle responses have not previously been investigated in DMD.

Anxiety disorders, particularly the fear-based disorders (such as social and separation anxiety, panic disorder and specific phobias) are associated with increased startle responses to unpredictable threat, which is thought to relate to hyperarousal and exaggerated anticipatory anxiety.^28,29^ Heightened reactivity to unpredictable threat has been implicated in the onset, maintenance and treatment response in fear-based anxiety disorders.^30,31^ Threat-induced anxiety symptoms can impact on executive skills, such as processing efficiency and cognitive flexibility, thus potentially affecting daily adaptive functioning.^32,33^ Anxiety symptoms are reported in 24–33% of people with DMD,^12,34,35^ and work from our group found increased fear-based anxiety symptoms (social and separation anxiety) in DMD compared to the typical population.^36^ Therefore, assessment of behavioural startle responses can provide an objective measure of anxiety.

Behavioural startle responses can be investigated using experimental fear-conditioning paradigms, where exposure to a threat stimulus causes an ‘innate’ or unconditioned physiological response. If a neutral stimulus is paired with the ‘threat’ stimulus, the physiological response becomes conditioned to occur with subsequent presentations of the neutral stimulus.^37^ We hypothesized that when tested using a fear-conditioning task, individuals with DMD would have heightened startle responses compared to control participants. We used a novel discrimination fear-conditioning task, which paired an aversive loud noise ‘threat’ stimulus with a neutral visual stimulus to elicit physiological startle responses that became conditioned to the neutral stimulus. These conditioned responses subsequently extinguished when the ‘threat’ was removed. Whilst this method of assessing psychophysiological responses is more suited to experimental than clinical settings, it provides both insight into the underlying neurobiological circuits involved in emotional disorders and the potential to measure the effects of therapeutic interventions.^31,38^ As an objective measure the startle response also has advantages over instruments more prone to subjectivity and bias, such as neuropsychological assessments and self-/informant-report neuropsychiatric symptom scales.^39,40^

Our aims were to determine whether group differences existed between DMD and control groups for the magnitude of unconditioned startle responses to threat, conditioned response acquisition and the degree of retention and extinction of conditioned responses. We also investigated associations of startle responses with anxiety and other neuropsychiatric symptoms and conducted an exploratory analysis of the influence of the Dp140 isoform on these outcomes.

## Materials and methods

### Study design and setting

This observational cross-sectional study compared startle responses in young males with DMD with an age-matched male control group using a fear-conditioning task. The study was conducted from February–_­_November 2019 at Great Ormond Street Hospital for Children (GOSH) and UCL Great Ormond Street Institute of Child Health.

### Participants

DMD and Control participants were recruited *via*: GOSH outpatient clinics (patients or siblings); a research database; advocacy groups; and advertising to UCL staff and students. Universal eligibility criteria were male gender, age 7–12 years and no significant visual or hearing impairment or noise-sensitivity. DMD-specific criteria were a genetic diagnosis of DMD, not receiving ataluren (dystrophin-modulating therapy that may cross the blood_­_–brain barrier); control-specific criteria were no neurological or psychiatric diagnosis. Informed written consent (parents) and assent (participants) was obtained according to the Declaration of Helsinki. All participants were allocated unique identifiers at recruitment. Ethical approval was granted by the Health Research Authority, London Bridge Research Ethics Committee (18.LO.1575).

Demographic and medical information was documented using a proforma: co-morbidities, medication, motor function, genotype (DMD), pubertal status and socio-economic status estimation.^41,42^

### Task parameters

**Figure 2:**
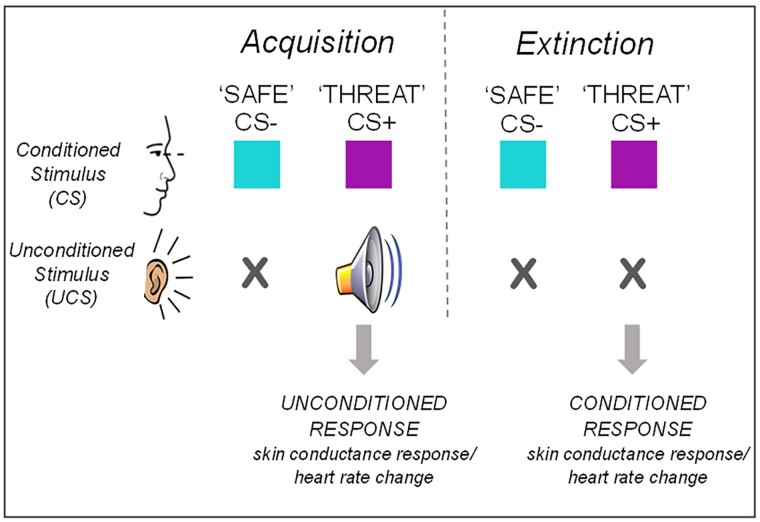
**Schematic representation of the fear-conditioning task.** Two different neutral stimuli, the CS, are presented to the subject in randomized trials during the response acquisition phase (‘Acquisition’). One is a ‘threat’ cue (CS+), which is paired with an aversive noise stimulus, the UCS. A ‘safe’ cue (CS−) is presented alone with no UCS. At the initial presentation of the UCS, behavioural and physiological responses, termed ‘unconditioned responses’, are elicited. With repeated presentation of trials in the Acquisition phase, a learned association develops between the CS+ cue and the unconditioned response, which then becomes ‘conditioned’. After 1–2 h, an Extinction phase is conducted in which both CS+ and CS− cues are presented, but with no UCS. The CS+ cue initially elicits the conditioned response, but after repeated trials not reinforced by the UCS, this association is extinguished and the CS+ cue no longer elicits the conditioned response, termed extinction. Prior to the Acquisition phase, a pre-task Calibration phase and Familiarization phase also occur, to allow physiological calibration manoeuvres, habituation to neutral stimuli and baseline measurements.

To determine physiological startle responses, we used a novel fear-conditioning task (Fig. 2) detailed in our previous work.^43^ Neutral visual conditioned stimuli (CS-, ‘safe’ cue and CS+, ‘threat’ cue) were coloured squares presented on a computer screen for 6 s. An aversive auditory unconditioned stimulus (UCS; white noise, approximately 85 dB) was presented binaurally through headphones with onset 5 s after CS+, duration 1 s and co-terminating with CS+. Four phases comprised: pre-task calibration; familiarization (CS+ and CS– presented with no aversive UCS; eight trials); acquisition (CS+ paired with UCS and CS– alone; three blocks of eight trials); and extinction (CS+ and CS– alone; five blocks of eight trials; occurring at least 1 h after acquisition). CS+/CS– order was pseudo-randomized, CS colour was randomly counterbalanced amongst participants and 10/12 CS+ trials were reinforced with UCS (∼80% reinforcement schedule).

Electrodermal activity (EDA) was recorded via surface skin electrodes on the non-dominant hand and ECG via standard three-lead electrodes. A Biopac MP160 unit recorded physiological parameters using AcqKnowledge 5.0.3 software (Biopac Systems, Inc., Aero Camino, CA).

Full-scale intelligence quotient (FSIQ) was assessed using the Wechsler Abbreviated Scale of Intelligence-II (WASI-II)^44^ that uses verbal and non-verbal reasoning tests without working memory and executive function tasks, which can be more impaired in DMD and thus could confound general IQ estimation.^45^ Participants’ parent/carers completed neuropsychiatric symptom questionnaires: anxiety (Screen for Child Anxiety Related Emotional Disorders, SCARED)^46^; internalizing and externalizing problems (Child Behavior Checklist, CBCL)^47^; social communication problems (Social Communication Disorder Checklist, SCDC)^48^; and inattention and hyperactivity (Conners’ Parent Rating Scale—Revised, CPRS-R).^49^ Several control group scores were higher than published population mean scores (FSIQ mean difference 14.0, *P* < 0.001); hyperactivity (*P* = 0.047) (Supplementary Table 1); therefore, we also compared DMD scores against age-/gender-matched normative data for each scale.^44,47,49–51^

DMD participants were stratified into subgroups determined by whether the mutation site predicted expression of shorter dystrophin isoforms: upstream of intron 44 predicts absent Dp427/intact Dp140 (‘Dp140+’); intron 44 to exon 50 predicts absent Dp427/indeterminate Dp140 expression (depending whether Dp140 promoter/long 5′UTR are affected; ‘Dp140_unk’); exon 51 to 62 predicts absent Dp427/Dp140 (‘Dp140−’); and exon 63 to 79 predicts absent Dp427/Dp140/Dp71 (‘Dp140−/71−’).

A subgroup of DMD participants (*n* = 11) repeated the fear-conditioning task after 3 months, confirming the test-retest reliability of the skin conductance response (results previously reported).^43^

### Data processing

A detailed description of data processing parameters is available in our previous work.^43^ EDA and ECG data were extracted from 0–12 s post-CS onset, and EDA alone from 0–6 s (First Interval Response, FIR) and 6–12 s (Second Interval Response, SIR) windows in acquisition to evaluate conditioned response acquisition and unconditioned response habituation, respectively. EDA represents the absolute skin conductance level (SCL) in microSiemens (μS), from which the skin conduction response (SCR) amplitude is derived: SCR = (SCL_max_)–(SCL_base_). SCL_base_ is baseline SCL at start of the trial, SCL_max_ is maximal SCL after CS onset. SCR metrics were defined for CS+ trials (SCR_CS+_), CS– trials (SCR_CS-_) and differential SCR, SCR_Diff_ = SCR_CS+_–SCR_CS–_ in contiguous trials. All SCRs were visually inspected for artefacts by a blinded researcher, cross-referencing digital event markers. Where artefacts affected data, responses were re-measured manually wherever possible, and if the SCR was completely obscured by artefacts, these data were excluded (see Maresh *et al*.^43^ for further details). All exclusions were reviewed independently by another researcher. Non-responders were defined as participants with SCR <0.01 µS in pre-task calibration manoeuvres and in ≥50% CS+/UCS trials, based on published recommendations.^52^ The non-responder rate was 1.9% (*n* = 1), lower than the quoted SCR non-responder rate of 10%.^53^

ECG data were transformed into R-R intervals (in seconds), the time interval between each heartbeat, where ‘R’ is the maximal positive peak of the ECG complex. Heart rate (HR) HR = 60/(R-R interval) beats per minute (bpm). Heart rate in CS+ trials = HR_CS+_; heart rate in CS– trials = HR_CS–_. Change in HR (ΔHR) = (mean HR in sampling window)—(mean baseline HR), where baseline is mean HR in the familiarization phase.

### Statistical considerations

The sample size (*n* = 56, DMD *n* = 31, controls *n* = 25) was determined primarily from previous literature and pragmatic considerations rather than formal power calculations, as there were no previous studies in this clinical population and measuring these outcomes. Previous similar studies have examined fear responses in anxious versus non-anxious children, with *n* = 35 (subgroups *n* = 17/18)^54^; *n* = 54 (subgroups *n* = 16/38)^55^; *n* = 60 (subgroups *n* = 8/11/19/16).^56^ As an additional guide from healthy paediatric data,^57^ a sample size of *n* = 56 (*n* = 28 per group) predicts 80% power to detect clinically important differences, although does not account for a healthy versus clinical population; therefore this pilot aimed to gather data in this population to guide future studies. Based on these considerations, we predicted the study had adequate power to detect significant differences in skin conductance responses for DMD versus control group comparisons, although was not powered to detect differences in the exploratory analyses of Dp140+ versus Dp140– subgroup comparisons.

Analysis was conducted by a researcher blinded to participants’ group and performed using IBM SPSSv.26, with a two-tailed significance level, alpha, of *P* = 0.05 and, where appropriate, Bonferroni adjustment for multiple comparisons.

Neuropsychiatric mean scores were compared with two-tailed independent samples *t*-tests. Chi-square tests compared task completion status. Analysis of block data used linear mixed model analysis, accounting for missing/unbalanced data, for (i) SCR_Diff_ and ΔHR startle response metrics (fixed effects = Group (DMD, control)/Block; random effect = StudyID; Group × Block pairwise comparisons); (ii) SCR_CS+_ and SCR_CS–_ in Extinction blocks (fixed effects = Group/Block/Stimulus (CS+/CS–); random effect = StudyID). Conditioned response extinction was defined as no significant discrimination between SCR_CS+_ and SCR_CS–_. The Residual Maximum Likelihood Estimation method was used with ‘Unstructured’ covariance structure.

IQ differed significantly between the DMD and Control groups (*P* < 0.001), Dp140+ and Dp140– groups (*P* = 0.03), and at the combined group level, IQ was significantly negatively correlated with anxiety, internalizing, externalizing, social communication and inattention symptoms, and moderately correlated with SCR_UC_ in the Control group (*rho* 0.39, *P* = 0.06). Therefore, to avoid confounding, we adjusted the analysis of primary outcomes for IQ (group differences in startle responses and association of startle responses with neuropsychiatric measures).

Between-groups univariate ANOVA evaluated primary physiological outcomes (unconditioned responses to first reinforced CS+ trial, SCR_UC_, ΔHR_UC_; conditioned SCR to first Extinction CS+ trial, SCR_EXT_), and within-groups repeated measures ANOVA evaluated ΔHR_UC_, taking IQ as a covariate. Between-group effect sizes, η^2^ were derived from univariate ANOVA output; within-group effect sizes were calculated from within-group general linear model analysis, where η^2^ = (treatment sum of squares)/(total sum of squares) with outcomes η^2^ ≥ 0.01 (small effect), η^2^ ≥ 0.06 (medium effect); and η^2^ ≥ 0.14 (large effect). Partial correlations (controlling for IQ) evaluated neuropsychiatric scores and startle response metrics associations.

A sensitivity analysis was conducted for primary outcome metrics to account for missing data, excluding ‘Non-completer’ participants who terminated the task early (completing <4/5 Extinction blocks) (Supplementary Table 2), showing no change in statistical significance for all the primary SCR metrics (unconditioned and conditioned SCR_CS+_) and an increase in significance for ΔHR_UC_, thus justifying use of the full dataset. A further sensitivity analysis for HR metrics excluded one DMD participant taking a beta-blocker (which can lower HR), which showed no difference in outcomes (Supplementary Table 2).

### Data availability

The authors confirm that the data supporting the findings of this study are available within the article and its Supplementary material. In view of the rarity of DMD and that genotype data is included, only aggregated data is available on reasonable request.

## Results

### Participants

Fifty-six participants (31 DMD and 25 Controls) took part in the study from the 63 initially recruited (Supplementary Fig. 1). Groups were age-matched [DMD 9.6 years (SD 1.4); Controls 9.7 years (SD 1.8)] and did not differ in mean pubertal stage or socio-economic status (Table 1). In the DMD group, 29/31 were ambulant (mean NorthStar score 21.2/34), 30/31 were taking corticosteroid treatment and 7/31 were taking cardiac medication as part of routine DMD treatment. Four DMD participants had diagnosed ASD and one ADHD.

**Table 1:** Participants’ baseline demographic information and available data for each group

	Control	DMD	DMD isoform subgroups			
			Dp140+	Dp140-	Dp140-/71-	Dp140_unk
**Demographics**						
Participants, *n*	25	31	12	11	1	7
Mean age, years (SD)	9.7 (1.8)	9.6 (1.4)	9.6 (1.6)	9.6 (1.5)	–	9.8 (1.1)
Non-ambulant, *n*	–	2	0	2	0	0
Mean NorthStar score (0–34)^a^	–	21.2	27.4	20.4	–	14.0
Cardiac medication, *n* (ACEi/BB)	–	ACEi *n* = 7; BB *n* = 1	ACEi *n* = 2; BB *n* = 1	ACEi *n* = 3; BB *n* = 0	ACEi *n* = 0; BB *n* = 0	ACEi *n* = 2; BB *n* = 0
Corticosteroid treatment, *n*	–	30 (D = 10; I = 19; A = 1)	11 (D = 8; I = 2; A = 1)	11 (D = 6; I = 5, A = 0)	1 (I = 1)	7 (D = 4; I = 3; A = 0)
Autism spectrum disorder diagnosis	0	4	0	4	0	0
ADHD diagnosis	0	2	0	0	1	1
Mean socio-economic status rank decile (1–10)^b^	6.4	7.1	7.8	7.4	–	5.8
Mean Pubertal Stage (1–5)^c^	1.6	1.5	1.3	1.6	–	1.6
**Available data**						
Parent-report neuropsychiatric scores, *n*	25	31	12	11	1	7
Participant self-report anxiety score, *n*	25	30	12	11	0	7
Intelligence quotient assessment, *n*	25	30	12	11	0	7
Skin conductance response data, *n*	24	28	10	11	1	6
HR data, *n*	25	31	12	11	1	7
Non-completers^d^, *n*	0	8	2	5	1	0

A = Alternate; ACEi = angiotensin converting enzyme inhibitor; BB = beta blocker; D = Daily; I = Intermittent.

NorthStar score is a 17-item lower limb functional assessment scale used in DMD, scored out of 34.

Socio-economic status mean decile score was derived from the Index of Multiple Deprivation for England^41^, available for participants living in England (*n* = 52; DMD = 28, Control = 24).

Pubertal stage was assessed using the parent/self-report ‘Growing and Changing’ questionnaire^42^. Scores range from 1 (pre-pubertal) to 5 (adult stage).

Non-completers were participants who terminated the task early, completing <4/5 extinction blocks.

Of the 56 physiological recording data sets, four were excluded from SCR analysis due to: physiological ‘Non-responder’ (DMD *n* = 1); protocol deviation (control *n* = 1); and technical equipment problems (DMD *n* = 2). No control participants requested early task termination compared to eight ‘Non-completer’ DMD participants (*P* = 0.007), of whom two were Dp140+ participants (2/12), five were Dp140– (5/11) and one was Dp140–/71– (1/1) (Table 1). There were no differences in other characteristics of the ‘Completer’ and ‘Non-completer’ groups (Supplementary Fig. 2 and Supplementary Table 3). Data exclusion due to artefacts affected 3.8% Control and 7.5% DMD Acquisition trials and 16.5% Control and 21.3% DMD Extinction trials (Supplementary Fig. 2).

### The impact of dystrophin deficiency on neuropsychological profile

#### Dp427 deficiency: DMD versus Controls

We evaluated emotional problems (anxiety and internalizing problems), neurodevelopmental features (IQ), social communication problems, inattention, hyperactivity and externalizing problems (Table 2). The DMD group IQ was 20–25 points lower than that of the Controls on all IQ measures, including full-scale IQ (FSIQ: DMD 90.5 versus Controls 115.4; *P* < 0.001), in line with work from our group and others.^11–13^ Control group FSIQ was higher than the population normative score (mean difference 14.0, *P* < 0.001), but DMD group FSIQ was also lower than age-matched normative data (mean difference −10.9, *P* < 0.001; Supplementary Table 1 and Supplementary Fig. 3).

**Table 2:** Neuropsychological assessment mean score comparisons between groups and subgroups

	DMD versus Control			DMD Dp140− versus Dp140+		
Neuropsychological assessment^a^	Raw score, mean difference (95% CI)	*t*	Sig.^b^, *P*	Raw score, mean difference (95% CI)	*t*	Sig.^b^, *P*
FSIQ	−24.9 (−32.5, −17.4)	−6.7	**<0**.**001**	−13.6 (−25.3, −1.9)	−2.4	**0.03**
Anxiety	5.4 (0.09, 10.8)	2.0	**0.046**	3.1 (−5.4, 11.5)	0.8	0.46
Internalizing problems	4.6 (1.2, 7.9)	2.7	**0.009**	4.3 (−1.5, 10.0)	1.6	0.14
Externalizing problems	6.5 (0.9, 12.2)	2.3	**0.02**	4.3 (−7.7, 16.3)	0.7	0.47
Social communication problems	4.8 (1.9, 7.8)	3.2	**0.003**	4.4 (−0.9, 9.6)	1.7	0.10
Inattention	2.0 (0.06, 4.0)	2.1	**0.**0**4**	3.9 (0.6, 7.2)	2.5	**0.**0**2**
Hyperactivity	−0.12 (−2.3, 2.1)	−0.1	0.91	3.4 (0.2, 6.7)	2.2	**0.**0**4**

FSIQ-4 assessed using WASI-II. The other neuropsychological/neuropsychiatric scores were obtained using SCARED-P, CBCL, SCDC and CPRS-R.

Between-group comparisons performed with independent samples *t*-tests, showing mean difference and 95% CI. Two-tailed significance calculated using alpha level of *P* = 0.05. *P*-values <0.05 are highlighted in bold.

Most neuropsychiatric co-morbidity scores were higher in the DMD group than Controls and population norms, including anxiety, internalizing, externalizing, social communication and inattention symptoms, and excepting only hyperactivity. Hyperactivity scores in both Control and DMD groups were higher than the typical population (Supplementary Table 1 and Supplementary Fig. 3).

In the combined cohort, there were weak-moderate significant correlations (*rho* = 0.3–0.4) between IQ and all neuropsychiatric symptom scores apart from hyperactivity (Supplementary Table 4), but not in the separate DMD and Control groups, apart from a significant negative correlation of inattention with IQ in the DMD group.

#### Dp427 and Dp140 deficiency: Dp140+ versus Dp140−

Neuropsychological assessment scores for isoform subgroups, stratified by involvement of the Dp140 isoform (Dp140+, *n* = 12, and Dp140−, *n* = 11) and uncertain Dp140 expression (Dp140_unk, *n* = 7), are shown in Supplementary Table 1 and Supplementary Fig. 3. Deficiency of Dp140 was associated with lower FSIQ (Dp140+ 96.4 versus Dp140− 82.8; *P* = 0.03). Compared with the population mean, Dp140+ FSIQ was not significantly different (mean difference −5.0, 95% CI −14.0, 4.0; *P* = 0.25), whilst Dp140_unk FSIQ was lower by 9.0 points (−13.7, −4.3; *P* = 0.003) and Dp140− FSIQ was lower by 18.6 points (−27.2, −10.0; *P* < 0.001). That the Dp140_unk FSIQ lies approximately in between Dp140+ and Dp140− FSIQ highlights that this is a mixed group regarding the expression of Dp140.

Inattention and hyperactivity scores were significantly higher in the Dp140− compared with the Dp140+ subgroup, and trends of higher anxiety and internalizing scores in Dp140− compared to Dp140+. The Dp140− subgroup also scored significantly higher compared with population means for both emotional problems (anxiety, internalizing problems) and neurodevelopmental problems (social communication problems, inattention and hyperactivity), whereas the Dp140+ subgroup had higher scores only in internalizing problems (Supplementary Fig. 3 and Supplementary Table 1). Internalizing problems encompass anxiety, depression and somatic symptoms, the latter of which can lead to higher scores in children with chronic physical conditions.^58^

### Impact of a loss of dystrophin on psychophysiological outcomes

#### Baseline physiological responses

##### DMD versus Control

**Figure 3:**
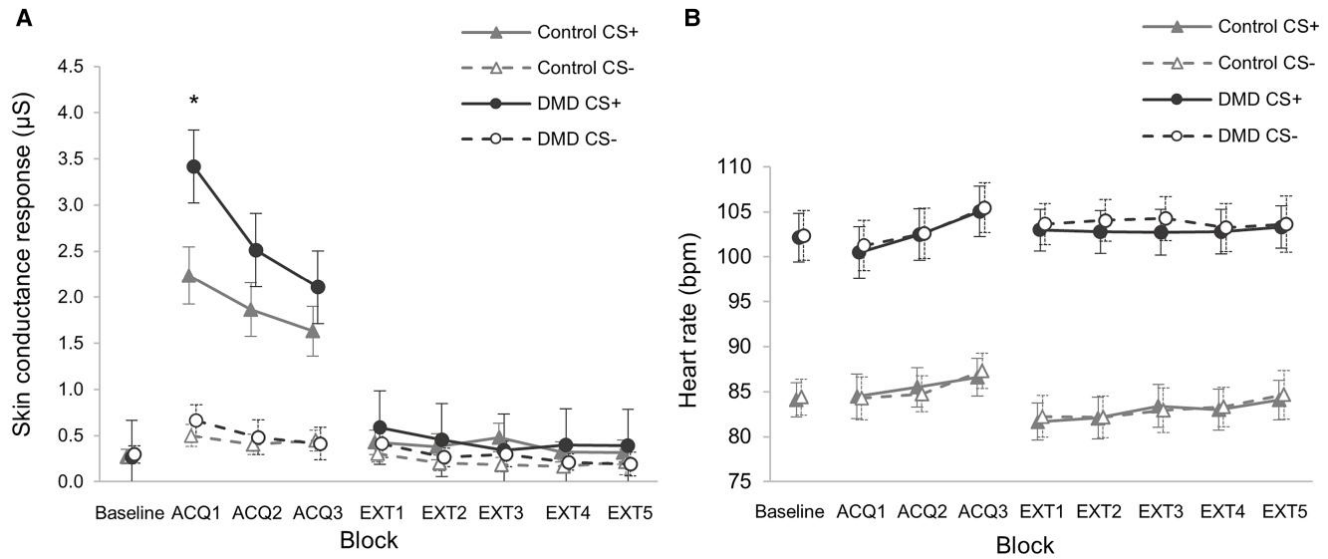
**Mean physiological responses to ‘threat’ and ‘safe’ conditioned stimuli (CS+ and CS−) by block for all fear conditioning task phases in DMD and Control groups.** (**A**) SCRS (measured in microSiemens, µS). Mean SCR in first Acquisition block (ACQ1) was significantly higher in the DMD compared to the Control group (*P = 0.03). (**B**) HR (bpm). SCR derived from EDA recorded from the palmar surfaces of digits 2 and 3, and defined as the baseline-to-peak EDA in the 12 s window following CS presentation. HR derived from the inter-beat interval from three-lead ECG recording. Error bars show the 95% CI.

Baseline data showed similar mean SCRs in DMD and Control groups [mean difference 0.008 µS (−0.18, 0.20), *P* = 0.93] and higher mean HR in the DMD group [mean difference 17.9 bpm (10.9, 24.8), *P* < 0.001] (Fig. 3 and Supplementary Fig. 4), consistent with the well-recognized phenomenon of resting sinus tachycardia in DMD.^59^

##### Dp140+ versus Dp140–

There was no difference in either mean baseline SCR and HR between the Dp140 + and Dp140− subgroups [baseline SCR mean difference 0.08 µS (−0.28, 0.44), *P* = 0.64; baseline HR mean difference −7.7 bpm (−19.0, 3.7), *P* = 0.18].

#### Unconditioned startle responses

##### DMD versus Control

**Figure 4:**
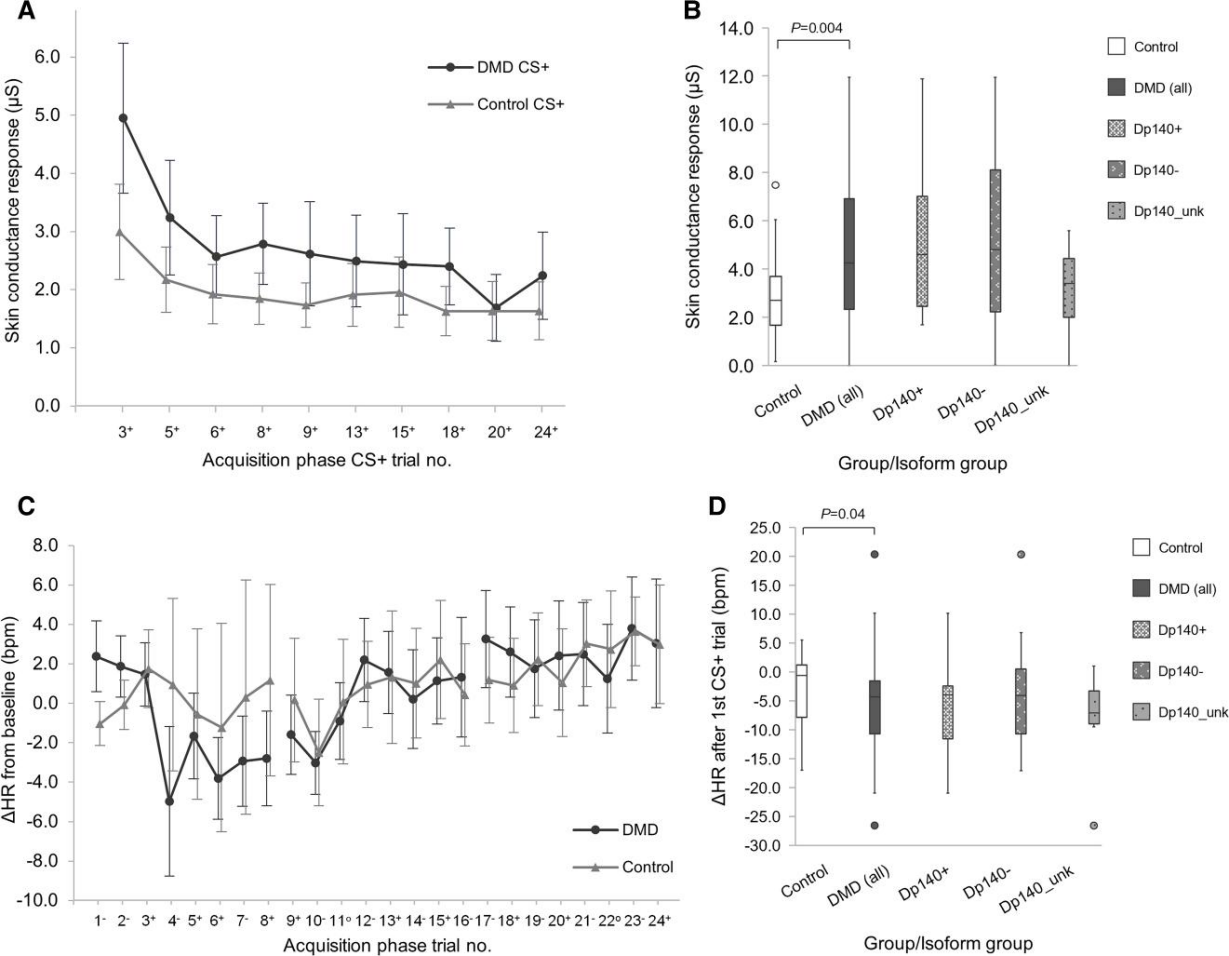
**Unconditioned responses to the aversive threat stimulus.** (**A**) SCR_CS+_ for DMD and Control groups in the Acquisition phase. Two ‘threat’ conditioned stimulus (CS+) trials were omitted (Acquisition trials 11 and 22) as these were unreinforced CS+ trials (no aversive noise presented). (**B**) Box plot of unconditioned SCR_CS+_ for the first CS+ trial (SCR_UC_) for Control, DMD and DMD isoform subgroups categorized by Dp140 isoform status. The Dp140+ group comprised DMD participants who retain the Dp140 isoform (*n* = 10); the Dp140**−** group comprised DMD participants who lack the Dp140- isoform (including Dp140**−** and Dp140**−**/71**−**; *n* = 12); and the Dp140_unk group contained DMD participants whose Dp140 status is uncertain (*n* = 6). (**C**) ΔHR from baseline (bpm) for DMD and Control groups for all Acquisition phase CS+ and CS**−** trials in the order as presented in the task, measured in bpm. ΔHR >0 indicates tachycardia relative to baseline; ΔHR <0 indicates bradycardia relative to baseline. The DMD group had a significant drop in HR from the first CS+ trial to the subsequent trial, the unconditioned ΔHR, ΔHR_UC_ (mean ΔHR_UC_**−**6.1 bpm; *P* = 0.006) but not the Control group (mean ΔHR_UC_**−**0.8 bpm; *P* = 0.99). (**D**) Box plot of ΔHR_UC_ showing a deceleration in HR in the DMD group but not the Control group, with a significant difference between groups in univariate analysis of variance: mean difference −8.7 bpm (95% CI −16.9, 0–0.51; *P* = 0.04). Error bars in line graphs indicate 95% CI (DMD, solid line; Control, dashed line). Solid bars in box plots indicate median values; error bars show the interquartile range calculated with inclusive median; outliers indicated with markers.

The mean unconditioned SCR to the initial threat stimulus (SCR_UC_) was higher in the DMD group compared with the Controls throughout the Acquisition phase (Fig. 3), most notably on the first CS+ trial [mean difference 3.0 µS (1.0, 5.1); *P* = 0.004; *η^2^* = 0.16] and first Acquisition block [mean difference 2.2 µS (0.9, 3.5), *P* = 0.001] (Table 3 and Fig. 4).

**Table 3:** Primary outcomes: skin conductance and HR response metrics in DMD group and DMD isoform subgroups compared with the Control group

Group	DMD versus Control			DMD Dp140 + versus Control			DMD Dp140− versus Control			DMD Dp140- versus Dp140+		
Outcome measure	Mean difference^d^ (95% CI)	*η^2^* ^e^	Sig.^f^, *P*	Mean diff. (95% CI)	*η^2^*	Sig., *P*	Mean difference (95% CI)	*η^2^*	Sig., *P*	Mean difference (95% CI)	*η^2^*	Sig., *P*
SCR_UC_ (µS)^a^	3.0 (1.0, 5.1)	0.16	**0.004**	3.4 (1.3, 5.6)	0.25	**0.003**	3.9 (1.1, 6.6)	0.20	**0.008**	0.61 (−3.0, 4.2)	0.01	0.73
ΔHR_UC_ (bpm)^b^	−8.7 (−16.9,−0.51)	0.08	**0.04**	−10.0 (−19.8,−0.19)	0.11	.**046**	−4.2 (−17.6, 9.1)	0.01	0.53	3.5 (−6.3, 13.2)	0.03	0.47
SCR_EXT_ (µS)^c^	0.37 (−0.23, 0.96)	0.03	0.22	0.34 (−0.21, 0.89)	0.05	0.22	−1.1 (−2.1, −0.21)	0.18	**0**.**02**	0.84 (−0.11, 1.8)	0.18	0.08

Bonferroni adjustment made for multiple comparisons. Covariate values for FSIQ = 101.9 for DMD versus Control; FSIQ = 109.5 for Dp140+ versus Control; FSIQ = 104.8 for Dp140− versus Control; FSIQ = 89.3 for Dp140− versus Dp140+. FSIQ data available for 30/31 DMD, 25/25 Control, 12/12 Dp140+ and 11/11 Dp140− participants.

SCR_UC_ = Unconditioned skin conductance response to the first ‘threat’ trial with conditioned stimulus, CS+, in microSiemens (µS).

ΔHR_UC_ = Unconditioned change in HR response to the first CS+ ‘threat’ trial (bpm).

SCR_EXT_ = Conditioned skin conductance response to the first CS+ trial of the Extinction phase (µS).

Adjusted mean difference from analysis of covariance pairwise comparisons including IQ as a covariate.

*η* ^2^ *= e*ffect size of Group.

Significance testing using univariate analysis of variance between groups/subgroups, adjusted for FSIQ, taking alpha of *P* = 0.05; *P*-values <0.05 are highlighted in bold.

The unconditioned change in HR after the initial threat stimulus, ΔHR_UC_, showed a fall in mean HR of 6.1 bpm in the DMD group (−10.7, −1.6; *P* = 0.006; *η^2^* = 0.04), but no change in the Control group [−0.8 bpm (−7.1, 5.8); *P* = 0.99; *η^2^* = 0.001] (Fig. 4). There was also a significant difference in ΔHR_UC_ between groups [mean difference −8.7 bpm (−0.51, −16.9); *P* = 0.04; *η^2^* = 0.08] (Table 3). This indicates a bradycardic response to threat in the DMD group only, which is more notable since heart rate variability is typically reduced in the DMD population.^60^

Due to the baseline group differences in IQ, all primary outcomes were adjusted for IQ (described in the 'Materials and methods' section). This adjustment did not affect the SCR_UC_ data findings, as significant group differences remaining after adjustment. ΔHR_UC_ group comparisons were only significant after adjustment. Unadjusted data are presented in Supplementary Table 5.

These data indicate that SCR_UC_ is effective in discriminating between the DMD and Control groups, and ΔHR_UC_ may be a useful secondary outcome.

##### Dp140+ versus Dp140–

There was no difference in unconditioned startle responses between Dp140+ and Dp140− subgroups [SCR_UC_ mean difference 0.61 µS (−3.0, 4.2), *P* = 0.73; ΔHR_UC_ mean difference 3.5 bpm (−6.3, 13.2), *P* = 0.47].

#### Acquisition of conditioned responses in both groups

**Figure 5:**
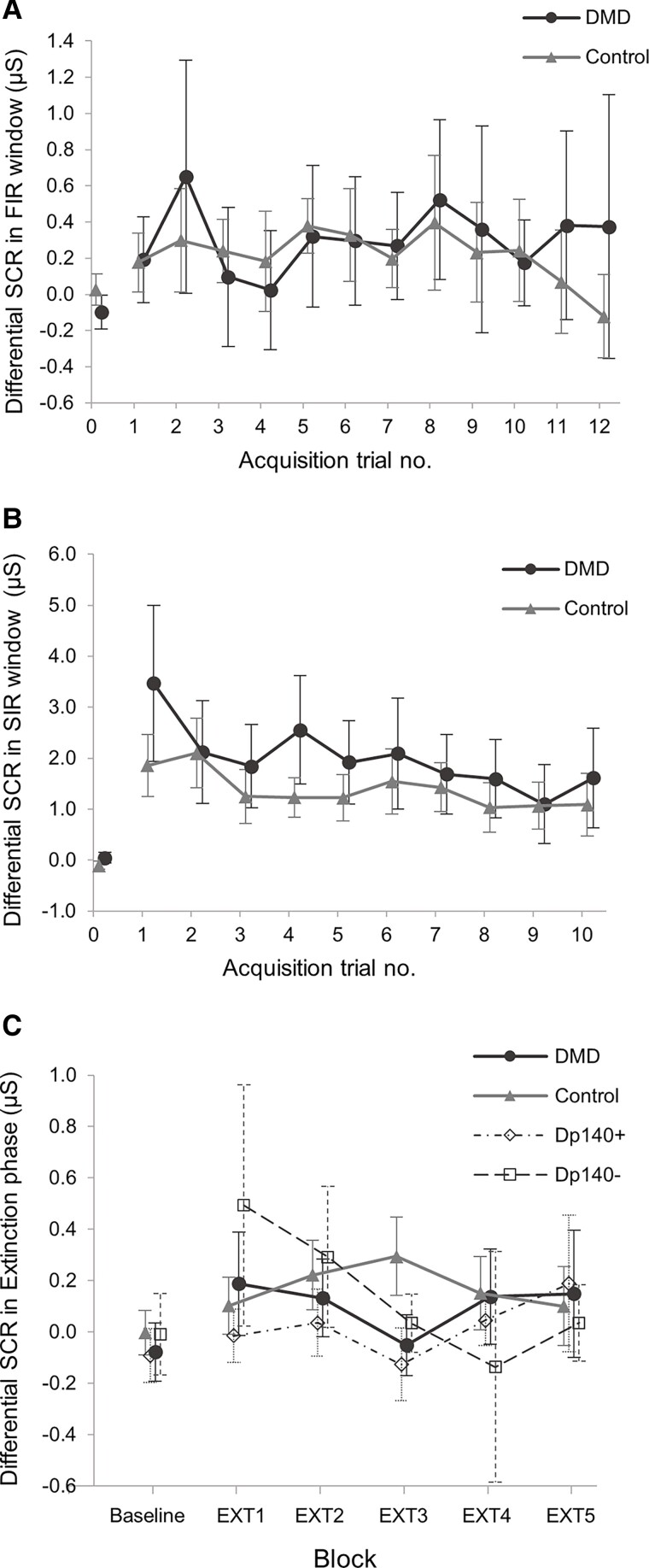
**Conditioned response acquisition, habitutation to unconditioned stimulus and retention of conditioned responses.** SCRs shown as the differential SCR (SCR_Diff_) to represent the degree of discrimination between the ‘threat’ conditioned stimulus (CS+) and ‘safe’ conditioned stimulus (CS−) cues. SCR_Diff_ = SCR_CS+_–SCR_CS−_ in contiguous trial pairs; SCR_Diff_ = 0 indicates no difference in response to the CS+ and CS− cues. (**A**) Mean SCR_Diff_ in the FIR window (0–6 s after CS onset) for the DMD and Control groups. This shows SCR_Diff_ after CS presentation but before the aversive noise, indicating the degree of learned response acquisition. For Acquisition trial 1, the FIR measurement occurred before participants had been presented with the first aversive stimulus, therefore this is lower than for the subsequent trials in both groups. (**B**) Mean SCR_Diff_ in the SIR window (6–12 s after CS onset, the start of which corresponds to the onset of the aversive ‘threat’ stimulus) for the DMD and Control groups. These are unconditioned responses to the aversive stimulus, which are shown to reduce or ‘habituate’ with repeated presentations of the aversive stimulus. For SIR data, one trial pair in each of Acquisition blocks 2 and 3 was omitted, as these included unreinforced CS+ trials (no aversive noise presented). (**C**) Mean SCR_Diff_ in the Extinction phase blocks for the Control, DMD and DMD isoform subgroups (Dp140+ and Dp140−). Extinction of the conditioned response (SCR_CS+_) is defined at the point at which there is no discrimination between the CS+ and CS+ cues, i.e. when SCR_Diff_ = 0. Extinction data were extracted from the whole response window (0–12 s after CS+ presentation). Mean SCR for familiarization phase is included as a baseline comparison in all plots. Error bars show 95% CI for each data point.

DMD and Control groups showed successful discrimination between ‘threat’ CS+ and ‘safe’ CS− cues in all Acquisition blocks, indicating that the paradigm was effective (Supplementary Table 6). Acquisition of the conditioned response was similar in both groups, with no difference in differential SCR between CS+ and CS− trials, SCR_Diff_, in the FIR window (Fig. 5A and Supplementary Table 6). This suggests similar fear learning in both groups. Habituation of the unconditioned response, indicating reduced salience of the threat stimulus with repeated presentations, was also seen in both DMD and Control groups with significant reduction in SCR_CS+_ from Acquisition block 1 to block 3 (Fig. 5B and Supplementary Table 6). Habituation is a well described phenomenon in fear-conditioning paradigms.^61–63^

#### Retention and extinction of conditioned skin conductance responses

##### DMD versus Control

The conditioned response was not successfully retained in either the DMD or Control groups at the start of the Extinction phase, with no discrimination between SCR_CS+_ and SCR_CS-_ in the first Extinction block in either group [DMD mean difference 0.18 µS (−0.1, 0.4), *P* = 0.21; Control mean difference 0.09 µS (−0.2, 0.4), *P* = 0.52] and no difference in SCR_EXT_ in the first Extinction trial between DMD and Control groups [mean difference 0.37 µS (−0.23, 0.96), *P* = 0.22] (Table 3), although there was a trend of completed extinction in the DMD group by the third block (Fig. 5C).

##### Dp140+ versus Dp140–

In contrast to the whole group data, the DMD Dp140− subgroup showed significant retention of the conditioned response with higher SCR_EXT_ compared to Controls [mean difference 1.1 µS (0.2, 2.1), *P* = 0.02, *η^2^* = 0.18] and a clear pattern of conditioned response extinction in Fig. 5C, neither of which were seen in the Dp140+ subgroup (Table 3). The difference in SCR_EXT_ between the Dp140+ and Dp140− subgroups was not statistically significant, however this may have been due to the small group sizes [mean difference 0.84 µS (−0.1, 1.8), *P* = 0.08, *η^2^* = 0.18]. Behavioural avoidance responses during Extinction were only observed in DMD participants: averting gaze during CS+ presentation and early termination in eight DMD boys (six Dp140 deficient). These findings suggest stronger conditioning in participants lacking the Dp140 isoform.

### Associations between unconditioned startle responses and the neuropsychiatric phenotype

Unconditioned physiological startle responses, SCR_UC_ and ΔHR_UC_, for the combined cohort correlated positively with Anxiety and Internalizing problems scores with SCR_UC_ (Anxiety versus SCR_UC_: *ρ* = 0.38, *P* = 0.01; Internalizing problems versus SCR_UC_: *ρ* = 0.33, *P* = 0.03; Supplementary Table 4). At the group/subgroup level the strongest correlations occurred for Anxiety versus SCR_UC_ in the Dp140− subgroup (*ρ* = 0.75, *P* = 0.01), with non-significant positive correlations in the other groups (DMD: *ρ* = 0.37, *P* = 0.08; Dp140+: *ρ* = 0.32, *P* = 0.40; Control: *ρ* = 0.22, *P* = 0.32). This may be due to a ‘floor effect’ from lower scores in the latter groups.

In contrast, neurodevelopmental problems scores for Inattention and Hyperactivity did not correlate with SCR_UC_, suggesting that this association is specific to anxiety alone and not related to more general psychopathology. Social communication problems scores showed positive but non-significant correlations in the combined cohort (*ρ* = 0.27, *P* = 0.07), most marked in the Dp140− subgroup (*ρ* = 0.54, *P* = 0.11), which may be a confounding effect of the well-established relationship between anxiety and social communication disorders.^64^

ΔHR_UC_ did not significantly correlate with any neuropsychiatric outcomes, although we noted moderate, non-significant correlations of ΔHR_UC_ with both Anxiety (*ρ* = 0.60, *P* = 0.07) and Internalizing problems (*ρ* = 0.46, *P* = 0.18) in the Dp140− group.

The positive correlations with Anxiety and Internalizing problems scores support the validity of the unconditioned skin conductance response, SCR_UC_, as a biological correlate for trait anxiety. These relationships were strongest for Dp140 deficient DMD participants, most likely reflecting the higher anxiety scores in this subgroup.

## Discussion

In recent years there has been an increasing understanding of the role that the multiple dystrophin isoforms have in brain function, both in human and various animal models.^11,12,65–67^ However, some aspects of the complex DMD neurobehavioural phenotype are only now beginning to be elucidated. There is growing evidence implicating dystrophin in fear and stress responses in experimental and naturally occurring animal models, including exaggerated startle responses in the *mdx* mouse,^19^ but until now there has been no systematic study of equivalent responses in humans with DMD. This study is the first to obtain objective evidence for a pathological unconditioned startle response in boys with DMD using a psychophysiological fear-conditioning task.

We found DMD participants had increased anxiety symptoms, with higher Anxiety and Internalizing problems scores compared to the Control group. Furthermore, the DMD subgroup lacking the Dp140 dystrophin isoform (Dp140−) had higher anxiety scores than the normal population, whilst the subgroup retaining the Dp140 isoform (Dp140+) did not. Previous studies have shown higher anxiety prevalence in DMD than the typical population, and whilst some observed that anxiety was greater in those with more distal 3′ mutations, in these studies the genotype stratifications did not allow determination of whether the Dp140 isoform was expressed or not.^11–13^ Recent work by Saoudi *et al.*^21^ showed that mice deficient in both Dp427 and Dp140 isoforms displayed increased anxiety compared to mice lacking only Dp427, further supporting our findings that associate Dp140 deficiency with a heightened anxiety phenotype.

Anxiety is the anticipation of a perceived future threat, associated with more long-lasting increased arousal and apprehension,^68^ whilst fear is a rapid-onset emotional response to an immediate threat, mediated by the amygdala, and well-conserved across vertebrate species.^38,69^ Human anxiety disorders are thought to be caused by excessive activation of innate fear circuits.^37^ In fear-conditioning tasks, anxious individuals typically show increased unconditioned startle responses compared to non-anxious individuals,^54,55^ and greater retention of conditioned responses in extinction.^28^

The psychophysiological responses we found in the DMD group have similarities with those seen in anxiety disorders; the DMD group had greater startle responses than Controls, most notably the unconditioned skin conductance response (SCR_UC_) but also the change in HR (ΔHR_UC_). SCR_UC_, but not ΔHR_UC_, also correlated with anxiety symptom scores (Anxiety and Internalizing problems) in the combined cohort, suggesting SCR_UC_ is a valid physiological correlate of trait anxiety.

Our study was designed to investigate the impact of loss of Dp427 dystrophin on the startle response in DMD, as Dp427 deficiency is a feature of all individuals with DMD. The increased startle responses we have demonstrated occurred for all genotypes of DMD, irrespective of whether the Dp140 isoform expression was also affected, and therefore can be attributed solely to deficiency of the full-length Dp427 isoform. This is consistent with animal studies: the *mdx* mouse, lacking only Dp427 dystrophin, displays an abnormal unconditioned startle response.^20^ We therefore conclude that the SCR_UC_ startle response is a useful biomarker of the deficiency of full-length Dp427 dystrophin.

In view of the known association of Dp140 deficiency with greater prevalence of neurodevelopmental problems,^11,12^ also confirmed in our cohort with lower IQ and increased inattention and hyperactivity symptoms, we also conducted an exploratory analyses comparing Dp140+ and Dp140− subgroups to investigate the influence of Dp140 on unconditioned startle responses and conditioned responses. Intriguingly, whilst there was no differential effect of the Dp140 isoform on the unconditioned startle response magnitude, the Dp140− subgroup showed increased retention of the conditioned SCR compared with Controls, which is a typical feature of anxiety disorders.^28,70^ Half of those lacking Dp140− terminated the Extinction phase early, which could be attributed to heightened arousal. Persistence of the conditioned response in Dp140− participants may also relate to increased salience of auditory stimuli, given the increased tendency to neurodevelopmental symptoms in this group that can be associated with sensory over-reactivity.^71^ These exploratory analyses were not powered to identify differences in startle responses between the isoform subgroups, therefore we cannot draw definite conclusions from the findings. However, our data indicate that while the deficiency of Dp427 is associated with increased anxiety prevalence and heightened activity in fear circuits, distal 3′ mutations that also affect Dp140 isoform expression may further exacerbate these symptoms. This is in line with recent studies in the *mdx52* mice lacking Dp140 as well as Dp427 demonstrating increased anxiety behaviours and pathophysiological differences between these mice and the *mdx* lacking only Dp427.^21,72^ Our observation warrants attention in future studies.

GABAergic synapse dysfunction has been demonstrated in the *mdx* mouse, lacking full length Dp427 dystrophin. In wild-type mice, GABA_A_-receptors (GABA_A_-R) on post-synaptic membranes co-localise with dystrophin at inhibitory synapses in the hippocampus, cerebellum, and amygdala.^73^ In the *mdx* mouse there is reduced clustering of GABA_A_-Rs and disrupted synaptic function in the basolateral amygdala and hippocampus.^20,73,74^ CNS dystrophin-restoration (either by direct injection or viral vector administration) of AONs^19,20,26,75,76^ corrects both the synaptic abnormalities and the exaggerated startle response. In a different mouse model with a heterozygous mutation in the γ_2_-GABA_A_-R subunit, reduced GABA_A_-receptor clustering also occurs and is associated with heightened responses in fear-conditioning tasks, increased harm avoidance behaviour and an explicit memory bias to threat cues.^77^ These findings suggest that reduced post-synaptic GABA_A_-R density is a key factor in the enhanced fear phenotype.

In typical humans, dystrophin is expressed in the so-called ‘limbic’ structures, including the amygdala, the hippocampal formation and the parahippocampal gyrus.^3,38,78^ Imaging studies have suggested limbic dysfunction occurs in DMD: hippocampal and medial temporal lobe hypometabolism has been observed by FDG-PET^79^; there is hippocampal hyperconnectivity in the default mode network fMRI^80^; GABA_A_-receptor distribution is abnormal in the prefrontal cortex.^22,79,80^ Dysfunction at GABAergic inhibitory synapses caused by loss of Dp427 dystrophin may therefore underlie the pathogenesis of at least some of the complex neuropsychiatric phenotype in DMD. Impaired GABAergic transmission has been implicated in the pathogenesis of autism spectrum disorder and depression.^81,82^ Accordingly, it is possible that some mental health issues associated with DMD are potentially reversible in humans following CNS-targeted dystrophin restoration, as in the *mdx* mouse.

The outlook for individuals with DMD now is very different to that in previous decades. Following improved standards of care, life-expectancy has increased by approximately 10 years,^83,84^ and genetic therapies addressing dystrophin deficiency in muscle are likely to further improve functional status and survival. However, the neuropsychiatric aspects of DMD impact on the daily functioning of individuals living with DMD and increase carer-burden.^36,85–87^ Current care recommendations advise consideration of neuropsychological referral at diagnosis of DMD, and if neurodevelopmental problems arise,^85^ although there is no specific guidance for assessment, monitoring or treatment of psychiatric disorders and specific treatment for psychiatric disorders in DMD is uncommon.^86^ Therefore, it is increasingly important to identify and manage neuropsychiatric symptoms with therapeutic interventions, and potentially with future disease-modifying CNS-acting therapies.

CNS-targeted intrathecal AON therapy is effective in spinal muscular atrophy,^88^ however in DMD systemic AON therapy is the primary requirement to ameliorate the muscle pathology. The current peripherally administered AONs in DMD do not cross the blood-brain barrier, but systemically delivered AONs with improved CNS penetration are under development,^26,89^ which could potentially enable dystrophin restoration in muscle and CNS from the same systemically administered therapy.

We acknowledge several limitations in this study. As this was the first study of its kind in this clinical population, sample sizes were primarily based on previous literature and pragmatic considerations as accurate *a priori* power calculations were not possible, although power estimates from healthy paediatric data supported our sample size. Isoform subgroup analyses were exploratory outcomes and subgroup sizes were underpowered, therefore the subgroup findings should be interpreted with caution, however as DMD is a rare disorder and some genotypes are uncommon it can be practically difficult to recruit to all genotype subgroups. We compared DMD participants against age- and sex-matched healthy control subjects in this proof-of-principle investigation, however we did not control for steroid use or physical disability, which could potentially be confounding factors. Data loss due to artefacts and early termination limited Extinction phase data interpretation, although had little impact on primary startle responses. Early termination of the task in ‘Non-completer’ participants may have been partly due to some DMD participants finding the task challenging, particularly those deficient in Dp140 dystrophin, therefore modifications to further adapt the task for the DMD population should be considered for future studies. Conditioned response retention may have been enhanced if the extinction task was delayed, as optimal retention occurs after 24 h^90^; however, for practical reasons all task phases were performed on the same day to avoid repeat reduce visits, which may be more difficult especially for DMD participants. Selection bias may have occurred in the Control group, given the higher FSIQ scores compared to the normal population, and in the DMD group a potential bias towards participants without significant cognitive or neurodevelopmental problems. The emotional and behavioural screening instruments used are not diagnostic measures and may have limitations in DMD,^91^ therefore caution should be taken in interpreting neuropsychiatric data.

In conclusion, we have demonstrated for the first time that boys with DMD show increased physiological startle responses to threat, using the principle of ‘back-translation’ from the exaggerated startle response phenotype in *mdx* mice. This has implications both for the further understanding of the neurobiology of DMD and clinical management. We propose that a lack of full-length Dp427 dystrophin leads to a dysfunctional fear system which predisposes individuals with DMD to develop anxiety disorders. In keeping with recent data in Dp140 deficient *mdx* mice,^21^ our data also provides some evidence that the lack of the Dp140 dystrophin isoform may further increase this risk. To date, anxiety in DMD has received little attention in both research and clinical practice therefore robust prevalence data are lacking, however given the increased risk of anxiety due to underlying neurobiological changes we suggest that young boys with DMD should be actively monitored for anxiety and emotional problems from an early age to enable support, interventions and treatment where appropriate. Further investigation of anxiety and other neuropsychiatric disorders is needed to inform clinical practice and, where necessary, refine clinical guidelines to optimize the care of young people with DMD. Our findings also have implications for translational research, as to date no CNS biomarker of dystrophin has been identified that could be used in a clinical trial. We propose that the unconditioned skin conductance startle response is a useful objective physiological biomarker that could be used in future clinical trials aimed at evaluating CNS dystrophin restoration, analogous to the *mdx* startle response as an outcome in preclinical studies of CNS-targeted antisense oligonucleotide therapies. The findings from this study may inform current clinical management of anxiety disorders in DMD, and potentially be a first step towards evaluating future CNS-disease modifying therapies.

## Supplementary Material

awac048_Supplementary_Data

## References

[1] Hoffman EP , Brown RH, Jr, Kunkel LM. Dystrophin: the protein product of the Duchenne muscular dystrophy locus. Cell. 1987;51(6):919–928. 3319190 10.1016/0092-8674(87)90579-4

[2] Muntoni F , Torelli S, Ferlini A. Dystrophin and mutations: one gene, several proteins, multiple phenotypes. Lancet Neurol. 2003;2(12):731–740. 14636778 10.1016/s1474-4422(03)00585-4

[3] Doorenweerd N , Mahfouz A, van Putten M, et al Timing and localization of human dystrophin isoform expression provide insights into the cognitive phenotype of Duchenne muscular dystrophy. Sci Rep. 2017;7(1):12575. 28974727 10.1038/s41598-017-12981-5 PMC5626779

[4] Falzarano M , Rachele R, Mietto M, et al Expression and localization of dystrophin isoforms transcripts in human adult control brain areas. Neuromuscul Disord. 2021;31:S89.

[5] Tadayoni R , Rendon A, Soria-Jasso LE, Cisneros B. Dystrophin Dp71: the smallest but multifunctional product of the Duchenne muscular dystrophy gene. Mol Neurobiol. 2012;45(1):43–60. 22105192 10.1007/s12035-011-8218-9

[6] Cotton S , Voudouris NJ, Greenwood KM. Intelligence and Duchenne muscular dystrophy: full-scale, verbal, and performance intelligence quotients. Dev Med Child Neurol. 2001;43(7):497–501. 11463183 10.1017/s0012162201000913

[7] Snow WM , Anderson JE, Jakobson LS. Neuropsychological and neurobehavioral functioning in Duchenne muscular dystrophy: a review. Neurosci Biobeha Rev. 2013;37(5):743–752. 10.1016/j.neubiorev.2013.03.016 23545331

[8] Moizard M-P , Billard C, Toutain A, et al Are Dp71 and Dp140 brain dystrophin isoforms related to cognitive impairment in Duchenne muscular dystrophy? Am J Med Genet. 1998;80(1):32–41. 9800909 10.1002/(sici)1096-8628(19981102)80:1<32::aid-ajmg6>3.0.co;2-y

[9] Daoud F , Angeard N, Demerre B, et al Analysis of Dp71 contribution in the severity of mental retardation through comparison of Duchenne and Becker patients differing by mutation consequences on Dp71 expression. Hum Mol Genet. 2009;18(20):3779–3794. 19602481 10.1093/hmg/ddp320

[10] Taylor PJ , Betts GA, Maroulis S, et al Dystrophin gene mutation location and the risk of cognitive impairment in Duchenne muscular dystrophy. PloS One. 2010;5(1):e8803. 20098710 10.1371/journal.pone.0008803 PMC2808359

[11] Ricotti V , Mandy WPL, Scoto M, et al Neurodevelopmental, emotional, and behavioural problems in Duchenne muscular dystrophy in relation to underlying dystrophin gene mutations. Dev Med Child Neurol. 2016;58(1):77–84. 26365034 10.1111/dmcn.12922

[12] Darmahkasih AJ , Rybalsky I, Tian C, et al Neurodevelopmental, behavioral, and emotional symptoms common in Duchenne muscular dystrophy. Muscle Nerve. 2020;61(4):466–474. 31909820 10.1002/mus.26803

[13] Colombo P , Nobile M, Tesei A, et al Assessing mental health in boys with Duchenne muscular dystrophy: Emotional, behavioural and neurodevelopmental profile in an Italian clinical sample. Eur J Paediatr Neurol.2017;21(4):639–647. 28392227 10.1016/j.ejpn.2017.02.007

[14] Sicinski P , Geng Y, Ryder-Cook AS, et al The molecular basis of muscular dystrophy in the mdx mouse: a point mutation. Science. 1989:244 (4912):1578–1580. 2662404 10.1126/science.2662404

[15] Muntoni F , Mateddu A, Serra G. Passive avoidance behaviour deficit in the mdx mouse. Neuromuscul Disord. 1991;1(2):121–123. 1822782 10.1016/0960-8966(91)90059-2

[16] Vaillend C , Billard J-M, Laroche S. Impaired long-term spatial and recognition memory and enhanced CA1 hippocampal LTP in the dystrophin-deficient Dmd(mdx) mouse. Neurobiol Dis. 2004;17(1):10–20. 15350961 10.1016/j.nbd.2004.05.004

[17] Remmelink E , Aartsma-Rus A, Smit AB, et al Cognitive flexibility deficits in a mouse model for the absence of full-length dystrophin. Genes Brain Behav. 2016;15(6):558–567. 27220066 10.1111/gbb.12301

[18] Miranda R , Nagapin F, Bozon B, et al Altered social behavior and ultrasonic communication in the dystrophin-deficient mdx mouse model of Duchenne muscular dystrophy. Mol Autism. 2015;6:60. 26527530 10.1186/s13229-015-0053-9 PMC4627616

[19] Vaillend C , Chaussenot R. Relationships linking emotional, motor, cognitive and GABAergic dysfunctions in dystrophin-deficient mdx mice. Hum Mol Genet. 2017;26(6):1041–1055. 28087735 10.1093/hmg/ddx013

[20] Sekiguchi M , Zushida K, Yoshida M, et al A deficit of brain dystrophin impairs specific amygdala GABAergic transmission and enhances defensive behaviour in mice. Brain. 2009;132(1):124–35. 18927146 10.1093/brain/awn253

[21] Saoudi A , Zarrouki F, Sebrie C, et al Emotional behavior and brain anatomy of the mdx52 mouse model of Duchenne muscular dystrophy. Dis Model Mech. 2021;14:9. 10.1242/dmm.049028 PMC8476816 34546327

[22] Suzuki Y , Higuchi S, Aida I, Nakajima T, Nakada T. Abnormal distribution of GABAA receptors in brain of Duchenne muscular dystrophy patients. Muscle Nerve. 2017;55(4):591–595. 27543743 10.1002/mus.25383

[23] Tozawa T , Itoh K, Yaoi T, et al The shortest isoform of dystrophin (Dp40) interacts with a group of presynaptic proteins to form a presumptive novel complex in the mouse brain. Mol Neurobiol. 2012;45(2):287–297. 22258561 10.1007/s12035-012-8233-5 PMC3311850

[24] Fritschy JM , Panzanelli P, Tyagarajan SK. Molecular and functional heterogeneity of GABAergic synapses. Cell Mol Life Sci. 2012;69(15):2485–2299. 22314501 10.1007/s00018-012-0926-4 PMC11115047

[25] Perronnet C , Vaillend C. Dystrophins, utrophins, and associated scaffolding complexes: role in mammalian brain and implications for therapeutic strategies. J Biomed Biotechnol. 2010;2010:849426. 20625423 10.1155/2010/849426 PMC2896903

[26] Goyenvalle A , Griffith G, Babbs A, et al Functional correction in mouse models of muscular dystrophy using exon-skipping tricyclo-DNA oligomers. Nat Med. 2015;21(3):270–275. 25642938 10.1038/nm.3765

[27] Verhaart IEC , Aartsma-Rus A. Therapeutic developments for Duchenne muscular dystrophy. Nat Rev Neurol. 2019;15(7):373–386. 31147635 10.1038/s41582-019-0203-3

[28] Duits P , Cath DC, Lissek S, et al Updated meta-analysis of classical fear conditioning in the anxiety disorders. Depress Anxiety. 2015;32(4):239–253. 25703487 10.1002/da.22353

[29] Gorka SM , Lieberman L, Shankman SA, Phan KL. Startle potentiation to uncertain threat as a psychophysiological indicator of fear-based psychopathology: An examination across multiple internalizing disorders. J Abnorm Psychol. 2017;126(1):8–18. 27868423 10.1037/abn0000233 PMC5215951

[30] Nelson BD , McGowan SK, Sarapas C, et al Biomarkers of threat and reward sensitivity demonstrate unique associations with risk for psychopathology. J Abnorm Psychol. 2013;122(3):662–671. 24016008 10.1037/a0033982 PMC3790143

[31] Gorka SM , Lieberman L, Klumpp H, et al Reactivity to unpredictable threat as a treatment target for fear-based anxiety disorders. Psychol Med. 2017;47(14):2450–2460. 28436351 10.1017/S0033291717000964

[32] Eysenck MW , Derakshan N, Santos R, Calvo MG. Anxiety and cognitive performance: attentional control theory. Emotion. 2007;7(2):336–353. 17516812 10.1037/1528-3542.7.2.336

[33] Cornwell BR , Mueller SC, Kaplan R, Grillon C, Ernst M. Anxiety, a benefit and detriment to cognition: behavioral and magnetoencephalographic evidence from a mixed-saccade task. Brain Cogn. 2012;78(3):257–267. 22289426 10.1016/j.bandc.2012.01.002 PMC3448553

[34] Banihani R , Smile S, Yoon G, et al Cognitive and Neurobehavioral Profile in Boys With Duchenne Muscular Dystrophy. J Child Neurol. 2015;30(11):1472–1482. 25660133 10.1177/0883073815570154

[35] Pangalila RF , van den Bos GA, Bartels B, et al Prevalence of fatigue, pain, and affective disorders in adults with Duchenne muscular dystrophy and their associations with quality of life. Arch Phys Med Rehabil. 2015;96(7):1242–1247. 25731937 10.1016/j.apmr.2015.02.012

[36] Trimmer RM K.E ; Muntoni F; Mandyo W. Understanding anxiety experienced by boys with Duchenne muscular dystrophy: A qualitative focus group study. In: British Paediatric Neurology Association Annual Meeting. Blackwell; 2020:60.

[37] Gross CT , Canteras NS. The many paths to fear. Nat Rev Neurosci. 2012;13(9):651–658. 22850830 10.1038/nrn3301

[38] LeDoux J . The emotional brain, fear, and the amygdala. Cell Mol Neurobiol. 2003;23(4–5):727–738. 14514027 10.1023/A:1025048802629 PMC11530156

[39] Kramer U , Grandjean L, Beuchat H. Emotions in clinical practice. In: Meiselman HL, editors. Emotion Measurement. 2nd edn. Woodhead Publishing; 2021:595–612.

[40] McDermott PA , Watkins MW, Rhoad AM. Whose IQ is it?–Assessor bias variance in high-stakes psychological assessment. Psychol Assess. 2014;26(1):207–214. 24188149 10.1037/a0034832

[41] Noble S MD , Noble M, Plunkett E, Gutacker N, Silk M, Wright G. The English Indices of Deprivation 2019. Secondary The English Indices of Deprivation 26th September 2019. https://www.gov.uk/government/statistics/english-indices-of-deprivation-2019

[42] Golding J . Growing and Changing: Parent and Son Questionnaire. Secondary Growing and Changing: Parent and Son Questionnaire 1999. http://www.bris.ac.uk/alspac/researchers/our-data/questionnaires/puberty-questionnaires/

[43] Maresh K , Papageorgiou A, Ridout D, et al Development of a novel startle response task in Duchenne muscular dystrophy. PLoS ONE. 2022; 17(4):e0264091. doi:10.1371/journal.pone.0264091 PMC9017900 35439255

[44] Wechsler DZ , Hsiao-pin C. WASI-II: Wechsler abbreviated scale of intelligence. Psychological Corporation; 2011.

[45] Battini R , Chieffo D, Bulgheroni S, et al Cognitive profile in Duchenne muscular dystrophy boys without intellectual disability: The role of executive functions. Neuromuscul Disord. 2018;28(2):122–128. 29305139 10.1016/j.nmd.2017.11.018

[46] Birmaher B , Khetarpal S, Brent D, et al The Screen for Child Anxiety Related Emotional Disorders (SCARED): scale construction and psychometric characteristics. J Am Acad Child Adolesc Psychiatry. 1997;36(4):545–553. 9100430 10.1097/00004583-199704000-00018

[47] Achenbach TM , Rescorla LA. Manual for the ASEBA school-age forms & profiles. University of Vermont, Research Center for Children, Youth, & Families; 2001.

[48] Constantino JN , Davis SA, Todd RD, et al Validation of a brief quantitative measure of autistic traits: comparison of the social responsiveness scale with the autism diagnostic interview-revised. J Autism Dev Disord. 2003;33(4):427–433. 12959421 10.1023/a:1025014929212

[49] Conners CK , Sitarenios G, Parker JD, Epstein JN. The revised Conners’ Parent Rating Scale (CPRS-R): factor structure, reliability, and criterion validity. J Abnorm Child Psychol. 1998;26(4):257–268. 9700518 10.1023/a:1022602400621

[50] Sequeira SL , Silk JS, Woods WC, Kolko DJ, Lindhiem O. Psychometric Properties of the SCARED in a Nationally Representative U. S. Sample of 5-12-Year-Olds. J Clin Child Adolesc Psychol. 2019;49(6):761–772. 31136197 10.1080/15374416.2019.1614001 PMC6881535

[51] Skuse DH , Mandy W, Steer C, et al Social communication competence and functional adaptation in a general population of children: preliminary evidence for sex-by-verbal IQ differential risk. J Am Acad Child Adolesc Psychiatry. 2009;48(2):128–137. 19106766 10.1097/CHI.0b013e31819176b8

[52] Lonsdorf TB Klingelhöfer-Jens M , Andreatta M, et al Navigating the garden of forking paths for data exclusions in fear conditioning research. Elife. 2019;8:e52465. 31841112 10.7554/eLife.52465 PMC6989118

[53] Braithwaite JJ , Watson D. G., Jones R., Rowe M., Guide for Analysing Electrodermal Activity (EDA) & Skin Conductance Responses (SCRs) for Psychological Experiments (Technical Report, version 2.0). Accessed 18 January 2017. https://www.birmingham.ac.uk/Documents/college-les/psych/saal/guide-electrodermal-activity.pdf

[54] Waters AM , Henry J, Neumann DL. Aversive Pavlovian conditioning in childhood anxiety disorders: impaired response inhibition and resistance to extinction. J Abnorm Psychol. 2009;118(2):311–321. 19413406 10.1037/a0015635

[55] Lau JYF , Lissek S, Nelson EE, et al Fear conditioning in adolescents with anxiety disorders: results from a novel experimental paradigm. J Am Acad Child Adoles Psychiatry. 2008;47(1):94–102. 10.1097/chi.0b01e31815a5f01 PMC2788509 18174830

[56] Jovanovic T , Nylocks KM, Gamwell KL, et al Development of fear acquisition and extinction in children: effects of age and anxiety. Neurobiol Learn Mem. 2014;113:135–142. 24183838 10.1016/j.nlm.2013.10.016 PMC4004724

[57] Gao Y , Raine A, Venables PH, Dawson ME, Mednick SA. The development of skin conductance fear conditioning in children from ages 3 to 8 years. Dev Sci. 2010;13(1):201–212. 20121876 10.1111/j.1467-7687.2009.00874.x PMC2818004

[58] Perrin EC , Stein RE, Drotar D. Cautions in using the Child Behavior Checklist: observations based on research about children with a chronic illness. J Pediatr Psychol. 1991;16(4):411–421. 1941423 10.1093/jpepsy/16.4.411

[59] Miller G , D’Orsogna L, O’Shea JP. Autonomic function and the sinus tachycardia of Duchenne muscular dystrophy. Brain Dev. 1989;11(4):247–250. 2774094 10.1016/s0387-7604(89)80044-0

[60] da Silva TD , Massetti T, Crocetta TB, et al Heart rate variability and cardiopulmonary dysfunction in patients with Duchenne muscular dystrophy: a systematic review. Pediatr Cardiol. 2018;39(5):869–883. 29696428 10.1007/s00246-018-1881-0

[61] Rescorla RA . Effect of US habituation following conditioning. J Comp Physiol Psychol. 1973;82(1):137–143. 4684968 10.1037/h0033815

[62] Dawson ME SA , Filion DL. The electrodermal system. In: Cacioppo JT, Tassinary LG, Berntson GG, ed. Handbook of psychophysiology. 3rd ed. Cambridge University Press; 2007.

[63] Boucsein W , Fowles DC, Grimnes S, et al Publication recommendations for electrodermal measurements. Psychophysiology. 2012;49(8):1017–1034. 22680988 10.1111/j.1469-8986.2012.01384.x

[64] Simonoff E , Pickles A, Charman T, et al Psychiatric disorders in children with autism spectrum disorders: prevalence, comorbidity, and associated factors in a population-derived sample. J Am Acad Child Adoles Psychiatry. 2008;47(8):921–929. 10.1097/CHI.0b013e318179964f 18645422

[65] Naidoo M , Anthony K. Dystrophin Dp71 and the neuropathophysiology of Duchenne muscular dystrophy. Mol Neurobiol. 2020;57(3):1748–1767. 31836945 10.1007/s12035-019-01845-w PMC7060961

[66] Comim CM , Ventura L, Freiberger V, et al Neurocognitive impairment in mdx mice. Mol Neurobiol. 2019;56(11):7608–7616. 31077034 10.1007/s12035-019-1573-7

[67] Chaussenot R , Edeline JM, Le Bec B, et al Cognitive dysfunction in the dystrophin-deficient mouse model of Duchenne muscular dystrophy: a reappraisal from sensory to executive processes. Neurobiol Learn Mem. 2015;124:111–1 22. 26190833 10.1016/j.nlm.2015.07.006

[68] Davis M , Walker DL, Miles L, Grillon C. Phasic vs sustained fear in rats and humans: role of the extended amygdala in fear vs anxiety. Neuropsychopharmacology. 2010;35(1):105–135. 19693004 10.1038/npp.2009.109 PMC2795099

[69] Mineka S , Ohman A. Phobias and preparedness: the selective, automatic, and encapsulated nature of fear. Biol Psychiatry. 2002;52(10):927–937. 12437934 10.1016/s0006-3223(02)01669-4

[70] Lenaert B , Boddez Y, Griffith JW, et al Aversive learning and generalization predict subclinical levels of anxiety: a six-month longitudinal study. J Anxiety Disord. 2014;28(8):747–753. 25254930 10.1016/j.janxdis.2014.09.006

[71] Cheung PPP , Siu AM. A comparison of patterns of sensory processing in children with and without developmental disabilities. Res Dev Disabil. 2009;30(6):1468–1480. 19665348 10.1016/j.ridd.2009.07.009

[72] Hashimoto Y KH , Sakai K, Fukushima Y, et al Brain Dp140 alters glutamatergic transmission and social behaviour in the mdx52 mouse model of Duchenne muscular dystrophy. Prog. Neurobiol. 2022;216:102288. 10.1016/j.pneurobio.2022.102288 35654209

[73] Knuesel I , Mastrocola M, Zuellig RA, et al Short communication: altered synaptic clustering of GABAA receptors in mice lacking dystrophin (mdx mice). Eur J Neurosci. 1999;11(12):4457–4462. 10594673 10.1046/j.1460-9568.1999.00887.x

[74] Vaillend C , Billard J-M. Facilitated CA1 hippocampal synaptic plasticity in dystrophin-deficient mice: role for GABAA receptors? Hippocampus. 2002;12(6):713–717. 12542223 10.1002/hipo.10068

[75] Dallerac G , Perronnet C, Chagneau C, et al Rescue of a dystrophin-like protein by exon skipping normalizes synaptic plasticity in the hippocampus of the mdx mouse. Neurobiol Dis. 2011;43(3):635–641. 21624465 10.1016/j.nbd.2011.05.012

[76] Vaillend C , Perronnet C, Ros C, et al Rescue of a dystrophin-like protein by exon skipping in vivo restores GABAA-receptor clustering in the hippocampus of the mdx mouse. Mol Ther. 2010;18(9):1683–1688. 20588257 10.1038/mt.2010.134 PMC2956917

[77] Crestani F , Lorez M, Baer K, et al Decreased GABAA-receptor clustering results in enhanced anxiety and a bias for threat cues. Nat Neurosci. 1999;2(9):833–839. 10461223 10.1038/12207

[78] Zhang W-H , Zhang J-Y, Holmes A, Pan B-X. Amygdala circuit substrates for stress adaptation and adversity. Biol Psychiatry. 2021;89(9):847–856. 33691931 10.1016/j.biopsych.2020.12.026

[79] Lee JS , Pfund Z, Juhasz C, et al Altered regional brain glucose metabolism in Duchenne muscular dystrophy: a PET study. Muscle Nerve. 2002;26(4):506–512. 12362416 10.1002/mus.10238

[80] Doorenweerd N , de Rover M, Marini-Bettolo C, et al Resting-state functional MRI shows altered default-mode network functional connectivity in Duchenne muscular dystrophy patients. Brain imaging Behav. 2021;15(5):2297–2307. 33389442 10.1007/s11682-020-00422-3 PMC8500880

[81] Pizzarelli R , Cherubini E. Alterations of GABAergic signaling in autism spectrum disorders. Neural Plast. 2011;2011:297153. 21766041 10.1155/2011/297153 PMC3134996

[82] Rudolph U , Mohler H. GABAA receptor subtypes: Therapeutic potential in Down syndrome, affective disorders, schizophrenia, and autism. Ann Rev Pharmacol Toxicol. 2014;54:483–507. 24160694 10.1146/annurev-pharmtox-011613-135947 PMC3997216

[83] Eagle M , Bourke J, Bullock R, et al Managing Duchenne muscular dystrophy–the additive effect of spinal surgery and home nocturnal ventilation in improving survival. Neuromuscul Disord. 2007;17(6):470–475. 17490881 10.1016/j.nmd.2007.03.002

[84] Birnkrant DJ , Bushby K, Bann CM, et al Diagnosis and management of Duchenne muscular dystrophy, part 1: diagnosis, and neuromuscular, rehabilitation, endocrine, and gastrointestinal and nutritional management. Lancet Neurol. 2018;17(3):251–267. 29395989 10.1016/S1474-4422(18)30024-3 PMC5869704

[85] Birnkrant DJ , Bushby K, Bann CM, et al Diagnosis and management of Duchenne muscular dystrophy, part 3: primary care, emergency management, psychosocial care, and transitions of care across the lifespan. Lancet Neurol. 2018;17(5):445–455. 29398641 10.1016/S1474-4422(18)30026-7 PMC5902408

[86] Hendriksen JGM , Thangarajh M, Kan HE, Muntoni F, group Etws. 249th ENMC International Workshop: The role of brain dystrophin in muscular dystrophy: Implications for clinical care and translational research, Hoofddorp, The Netherlands, November 29th-December 1st 2019. Neuromuscul Disord. 2020;30(9):782–794. 10.1016/j.nmd.2020.08.357 32912717

[87] Setchell J , Thille P, Abrams T, et al Enhancing human aspects of care with young people with muscular dystrophy: Results from a participatory qualitative study with clinicians. Child Care Health Dev. 2018;44(2):269–277. 29119577 10.1111/cch.12526

[88] Finkel RS , Mercuri E, Darras BT, et al Nusinersen versus sham control in infantile-onset spinal muscular atrophy. N Engl J Med. 2017;377(18):1723–1732. 29091570 10.1056/NEJMoa1702752

[89] Tsoumpra MK , Fukumoto S, Matsumoto T, et al Peptide-conjugate antisense based splice-correction for Duchenne muscular dystrophy and other neuromuscular diseases. EBioMedicine. 2019;45:630–645. 31257147 10.1016/j.ebiom.2019.06.036 PMC6642283

[90] Maren S . Nature and causes of the immediate extinction deficit: a brief review. Neurobiol Learn Mem. 2014;113:19–24. 24176924 10.1016/j.nlm.2013.10.012 PMC4004759

[91] Hellebrekers DMJ , Lionarons JM, Faber CG, et al Instruments for the assessment of behavioral and psychosocial functioning in Duchenne and becker muscular dystrophy; a systematic review of the literature. J Pediatr Psychol. 2019;44(10):1205–1223. 31429914 10.1093/jpepsy/jsz062

