# Supplementary material for: Startle responses in Duchenne muscular dystrophy: a novel biomarker of brain dystrophin deficiency
Source: Brain. 2022 Feb 8;146(1):252–65. doi: 10.1093/brain/awac048 (PMC9825594; doi:10.1093/brain/awac048)
Supplement: awac048_Supplementary_Data [file awac048_supplementary_data.pdf]

Supplementary Table 1. Neuropsychological assessment mean scores and group comparisons

		Between group comparisons[b]					Group vs. normative data comparisons[c]			
**Neuropsychological assessment[a]**	**Group**	**n**	**Mean raw score *(SD)***	**Mean difference *(95% CI)***	***t***	**Sig., *P***	**Normative data mean score *(SD)***	**Mean difference *(95% CI)***	***t***	**Sig., *P***
**Full Scale Intelligence Quotient (FSIQ)**	**Control**	25	115.4 *(14.4)*	-24.9 *(-32.5,-17.4)*	-6.7	**<.001**	101.4 *(13.0)*	14.0 *(8.0, 19.9)*	4.9	**<.001**
	**DMD**	30	90.5 *(13.2)*					-10.9 *(-15.8,-6.0)*	-4.5	**<.001**
	**DMD_Dp140+**	12	96.4 *(14.1)*	-13.6 *(-25.3,-1.9)*	-2.41	**.03**		-5.0 *(-14.0,4.0)*	-1.2	.25
	**DMD_Dp140-**	11	82.8 *(12.8)*					-18.6 *(-27.2,-10.0)*	-4.8	**<.001**
	**DMD_Dp140_unk**	7	92.4 *(5.1)*					-9.0 *(-13.7, -4.3)*	-4.7	**.003**
**Verbal Comprehension Index (VCI)**	**Control**	25	113.5 *(13.2)*	-21.4 *(-28.4,-14.3)*	-6.1	**<.001**	101.2 *(12.7)*	12.3 *(6.9, 17.7)*	4.7	**<.001**
	**DMD**	30	92.2 *(12.6)*					-9.0 *(-13.8,-4.3)*	-3.9	**<.001**
	**DMD_Dp140+**	12	97.3 *(12.9)*	-13.3 *(-23.9,-2.8)*	-2.6	**.02**		-3.9 *(-12.1, 4.3)*	-1.0	.32
	**DMD_Dp140-**	11	84.0 *(11.4)*					-17.2 *(-24.9,-9.5)*	-5.0	**<.001**
	**DMD_Dp140_unk**	7	96.1 *(7.4)*					-5.1 *(-11.9, 1.8)*	-1.8	.12
**Performance Reasoning Index (PRI)**	**Control**	25	113.8 *(15.8)*	-23.1 *(-4.2,-31.4)*	-5.5	**<.001**	101.2 *(12.5)*	12.6 *(6.1, 19.2)*	4.0	**<.001**
	**DMD**	30	90.8 *(15.0)*					-10.4 *(-16.0,-4.8)*	-3.8	**<.001**
	**DMD_Dp140+**	12	95.9 *(16.9)*	-10.8 *(-24.8,3.1)*	-1.6	.12		-5.3 *(-16.0, 5.4)*	-1.1	.30
	**DMD_Dp140-**	11	85.1 *(15.2)*					-16.1 *(-26.3,-5.9)*	-3.5	**<.001**
	**DMD_Dp140_unk**	7	90.9 *(8.5)*					-10.3 *(-18.2, -2.5)*	-3.2	**.02**

**Anxiety score**	**Control**	25	12.0 *(8.1)*	5.4 *(0.09, 10.8)*	2.0	**.046**	12.1 *(12.4)*	-0.06 *(-3.4, 3.3)*	-0.04	.97
	**DMD**	31	17.5 *(11.2)*					5.4 *(1.3, 9.5)*	2.7	**.01**
	**DMD_Dp140+**	12	16.7 *(11.3)*	3.1 *(-5.4, 11.5)*	0.75	.46		4.6 *(-2.6, 11.7)*	1.4	.19
	**DMD_Dp140-**	11	19.7 *(7.7)*					7.6 *(2.5, 12.8)*	3.3	**.008**
	**DMD_Dp140_unk**	7	17.3 *(15.8)*					5.2 *(-9.4, 19.8)*	0.8	.42
**Internalising problems**	**Control**	25	6.5 *(4.7)*	4.6 *(1.2, 7.9)*	2.7	**.009**	5.1 *(4.8)*	1.4 *(-0.6, 3.3)*	1.5	.16
	**DMD**	31	11.0 *(7.3)*					5.9 *(3.3, 8.6)*	4.6	**<.001**
	**DMD_Dp140+**	12	8.8 *(4.5)*	4.3 *(-1.5, 10.0)*	1.6	.14		3.7 *(0.8, 6.5)*	2.8	**.02**
	**DMD_Dp140-**	11	13.0 *(7.9)*					7.9 *(2.6,13.2)*	3.3	**.008**
	**DMD_Dp140_unk**	7	13.0 *(9.5)*					7.9 *(-0.8, 16.6)*	2.2	.07
**Externalising problems**	**Control**	25	5.5 *(7.1)*	6.5 *(0.9, 12.2)*	2.3	**.02**	6.6 *(6.0)*	-1.1 *(-4.0, 1.8)*	-0.8	.45
	**DMD**	31	12.1 *(12.4)*					5.5 *(0.9, 10.0)*	2.4	**.02**
	**DMD_Dp140+**	12	9.9 *(8.6)*	4.3 *(-7.7, 16.3)*	0.7	.47		3.3 *(-2.2, 8.8)*	1.3	.21
	**DMD_Dp140-**	11	14.2 *(17.9)*					7.6 *(-4.4, 19.6)*	1.4	.19
	**DMD_Dp140_unk**	7	12.3 *(9.4)*					5.7 *(-3.0, 14.4)*	1.6	.16
**Social communication problems**	**Control**	25	3.6 *(4.6)*	4.8 *(1.9, 7.8)*	3.2	**.003**	3.3 *(4.2)*	0.3 (-1.6, 2.2)	0.3	.75
	**DMD**	30	8.4 *(6.4)*					5.1 *(2.8, 7.5)*	4.4	**<.001**
	**DMD_Dp140+**	12	6.2 *(5.2)*	4.4	1.7	.10		2.9 *(-0.5, 6.2)*	1.9	.08

	**DMD_Dp140-**	11	10.6 *(6.9)*	*(-0.9, 9.6)*				7.2 *(2.6, 11.9)*	3.5	**.006**
	**DMD_Dp140_unk**	7	9.0 *(7.0)*					5.7 *(-0.8, 12.2)*	2.2	.07
**Inattention**	**Control**	25	4.1 *(3.2)*	2.0 *(0.06, 4.0)*	2.1	**.04**	3.0 *(2.8)*	1.1 *(-1.9, 2.4)*	1.8	.09
	**DMD**	31	6.2 *(4.0)*					3.2 *(1.7, 4.6)*	4.4	**<.001**
	**DMD_Dp140+**	12	4.1 *(3.8)*	3.9 *(0.6, 7.2)*	2.5	**.02**		1.1 *(-1.3, 3.5)*	1.0	.34
	**DMD_Dp140-**	11	8.0 *(3.9)*					5.0 *(2.4, 7.6)*	4.3	**.002**
	**DMD_Dp140_unk**	7	6.7 *(3.8)*					3.7 *(0.2, 7.2)*	2.6	**.04**
**Hyperactivity**	**Control**	25	4.3 *(4.3)*	-0.12 *(-2.3, 2.1)*	-0.1	.91	2.5 *(2.9)*	1.8 *(0.02, 3.5)*	2.1	**0.047**
	**DMD**	31	4.2 *(3.9)*					1.7 *(0.2, 3.1)*	2.4	**0.02**
	**DMD_Dp140+**	12	2.7 *(2.8)*	3.4 *(0.2, 6.7)*	2.2	**.04**		0.2 *(-1.6, 2.0)*	0.2	0.84
	**DMD_Dp140-**	11	6.1 *(4.6)*					3.6 *(0.5, 6.7)*	2.6	**0.03**
	**DMD_Dp140_unk**	7	3.6 *(3.7)*					1.1 *(-2.3, 4.5)*	0.8	.47

[a] Neuropsychological/neuropsychiatric assessments using either direct assessment (FSIQ/VCI/PRI), parent-report or self-report (Anxiety only) questionnaires: FSIQ-4/VCI/PRI - Wechsler Abbreviated Intelligence Scale – 2nd Edition; Anxiety - Screen for Child Anxiety Related Disorders Parent-report; Internalising and Externalising problems - Child Behavior Checklist (CBCL); Social communication problems - Social Communication Disorders Checklist; Inattention and Hyperactivity - Conners' Parent Rating Scale-Revised, short version.

[b] Between group comparisons performed with independent samples *t*-tests, showing mean difference, standard deviation (SD) and 95% confidence interval of the difference (95% CI); *t* test statistic. Sig. = Two-tailed significance, using alpha level of *P*=.05. *P*-values <.05 are highlighted in bold

[c] Group data compared to published age-matched population normative data for each instrument using one-sample *t*-tests. FSIQ-4/VCI/PRI normative data from WASI-II manual (Wechsler, D. (1999). Wechsler Abbreviated Scale of Intelligence. San Antonio, TX: The Psychological Corporation); Internalising/Externalising normative data from CBCL manual (Achenbach & Rescorla, 2001); Screen for Child Anxiety Related Disorders (SCARED) normative data from a large US population study (Sequeira et al, 2019); Social Communication Disorders Checklist normative data from a large UK population study (Skuse et al 2009); Conners' Parent Rating Scale-Revised normative data from published manual (Conners CK, Sitarenios G, Parker JDA, Epstein JN. The revised Conners' Parent Rating Scale (CPRS-R): factor structure, reliability, and criterion validity. Journal of Abnormal Child Psychology 1998;26(4):257-68). Normative data was available for: mean raw score, standard deviation (except SCARED) and sample size; males only (except SCARED which includes males and females); and was age-matched, except SCARED (11-12 years only) and SCDC (7-8 years only).

Supplementary Table 2. Sensitivity analyses of primary outcomes, accounting for (A) non-completers, and (B) DMD participants taking B-blockers.

**A Sensitivity analysis including/excluding non-completers**[a]									
	**Mean differences *(95% Confidence Intervals)* for between groups comparisons**[b] **(DMD vs. Control)**								
	**n**	**SCR_{UC}, *μS***[c]	**Sig., *P***	**SCR_{CS+} ACQ1 block, *μS***[d]	**Sig., *P***	**SCR_{EXT}, *μS***[e]	**Sig., *P***	**ΔHR_{UC}, *bpm***[f]	**Sig., *P***
DMD all	28	3.1 *(1.0, 5.3)*	**.005**	2.3 *(1.0, 3.6)*	**.001**	0.37 *(-0.23, 0.96)*	0.2	-8.8 (-17.2, -0.3)	**.04**
DMD excl. non-completers	20	3.5 *(1.3, 5.6)*	**.002**	2.6 *(1.2, 3.9)*	**<.001**	0.31 *(-0.3, 0.9)*	0.30	-9.5 (-18.4, -0.6)	**.04**

**B Sensitivity analysis including/excluding B-blocker participant**[b]					
		**ΔHR_{UC} within groups, in bpm**		**Mean difference in ΔHR_{UC} *(95% Confidence Intervals)*, in bpm, between DMD vs. Control**	
	n	Mean ΔHR_{UC} *(sd)*	Sig., *P*, within group	Mean diff. ΔHR_{UC} *(95%CI)*	Sig., *P* (DMD vs. Control)
Control	25	-0.8 *(12.7)*	.75		
DMD all	31	-6.4 *(9.7)*	**.001**	-8.7 *(-17.0, -0.5)*	**.04**
DMD excl. B-blockers	30	-6.6 *(9.9)*	**.001**	-9.1 *(-17.5, -0.7)*	**.03**

[a] Non-completers were defined as participants completing <4 extinction blocks. All primary SCR and HR metrics were derived prior to this time point.
[b] Significance (Sig.) testing using univariate ANOVA adjusted for IQ for between-groups analyses, and repeated measures ANOVA for within-group analyses. Alpha level *P*=.05. *P*-values <.05 are highlighted in bold. Bonferroni adjustment made for multiple comparisons.
[c] SCR_{UC} = Unconditioned skin conductance response to the first 'threat' trial with conditioned stimulus, CS+, in microSiemens (μS).
[d] SCR_{CS+} ACQ1 = Skin conductance response to 'threat' CS+ trials in first Acquisition block.
[e] SCR_{EXT} = Conditioned skin conductance response to the first CS+ trial of the Extinction phase, in μS.
[f] ΔHR_{UC} = Unconditioned change in heart rate response to the first CS+ 'threat' trial, in beats per minute (bpm).

Supplementary Figure 1. Flow Diagram of study recruitment.

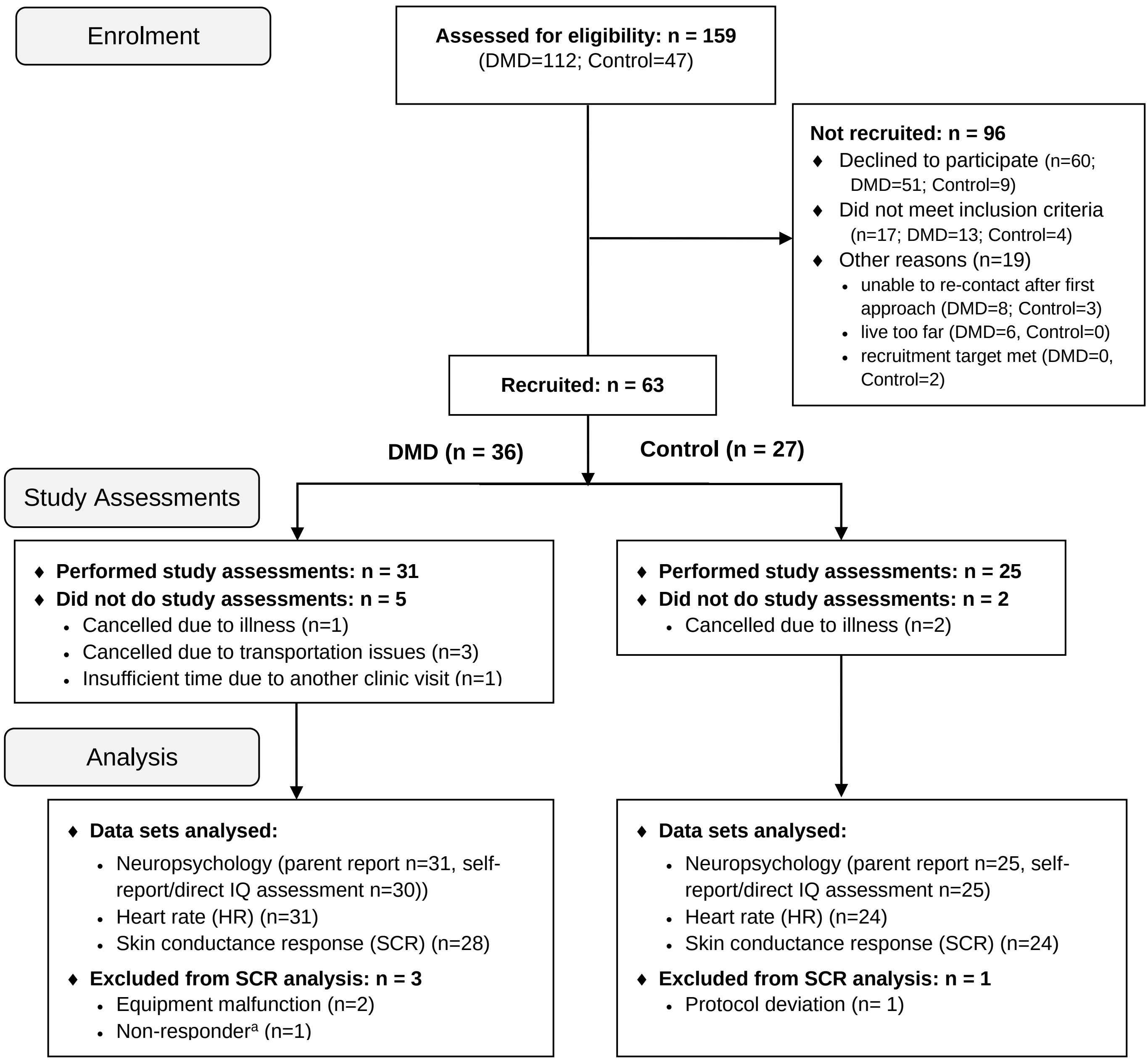

[a]Non-responders in skin conductance recordings were defined as participants in whom ≥ 50% raw SCRs following UCS presentations and pre-exposure tasks were <0.01 μS, based on recommendations from previous literature (Lonsdorf et al, 2019; Marin et al 2019; Hu et al 2019).

Supplementary Figure 2

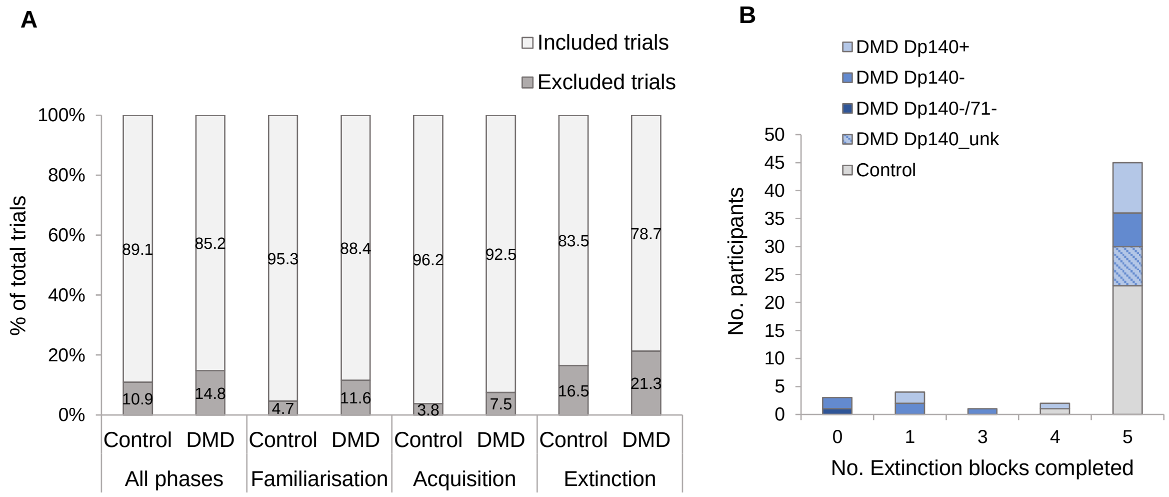

Supplementary Figure 2. Data exclusion and drop-out.

(**A**) The percentage of individual SCR trial data excluded from analysis due to artefacts. (**B**) No. of Extinction blocks (8 trials each) completed by DMD (n=28) and Control (n=24) participants included in SCR analysis. DMD group is split into isoform subgroups. 24/24 (100%) of Control and 20/28 (71.4%) DMD participants completed 4 or more extinction blocks. Of the 8 DMD 'Non-completers' (completing <4 blocks, 6/8 (75%) lacked the Dp140 isoform (5 Dp140-; 1 Dp140-/71-) and 2/8 retained it (Dp140+).

Supplementary Table 3. Demographics and baseline neuropsychiatric data for DMD completers and non-completers in extinction phase.

	**Completer**[a]	**Non-completer**[a]		
Median no. extinction blocks completed *(range)*	5 *(4, 5)*	1 *(0,3)*		
Median no. extinction trials completed (range)	40.0 *(40, 40)*	9.5 *(0, 24)*		
**Participants**	**n (%)**	**n (%)**		
Total DMD participants	23 *(74.2)*	8 *(25.8)*		
Dp 140+, n	10 (83.3)	2 (16.7)		
Dp140-, n[a]	6 (50.0)	6 *(50.0)*		
Dp140_unk, n	7 (100.0)	0 *(0)*		
**Demographics and baseline neuropsychiatric data**	**Median *(range)***	**Median *(range)***	***U***[d]	***Sig., P***
Age, years	9.84 *(7.4, 12.0)*	9.3 *(7.1, 11.4)*	74.0	.44
Full scale IQ[b]	92 *(59, 120)*	86 *(71, 100)*	52.5	.17
Anxiety Raw score	17.0 *(0, 46)*	14.5 *(4, 33)*	78.0	.94
Internalising Raw score	8.0 *(3, 32)*	8.5 *(3, 26)*	75.0	.46
Externalising Raw score	9.0 *(1, 29)*	9.0 *(0, 20)*	79.0	.58
SCDC Total score	7.0 *(0, 19)*	4.0 *(0, 22)*	75.0	.46
Inattention Raw score	5.0 *(0, 14)*	8.0 *(2, 11)*	115.0	.32
Hyperactivity Raw score	3.0 *(0, 14)*	3.5 *(0, 10)*	132.0	.88

[a] 'Completer' is a participant completing ≥ 4 blocks in Extinction phase; 'Non-completer' is a participant completing < 4 Extinction blocks.
[b] Dp140- group includes Dp140-/71-
[c] For FSIQ in non-completers n=7.
[d] Significance (Sig.) testing for group comparisons conducted with Mann-Whitney U-test, taking alpha level *P*=.05.

Supplementary Figure 3

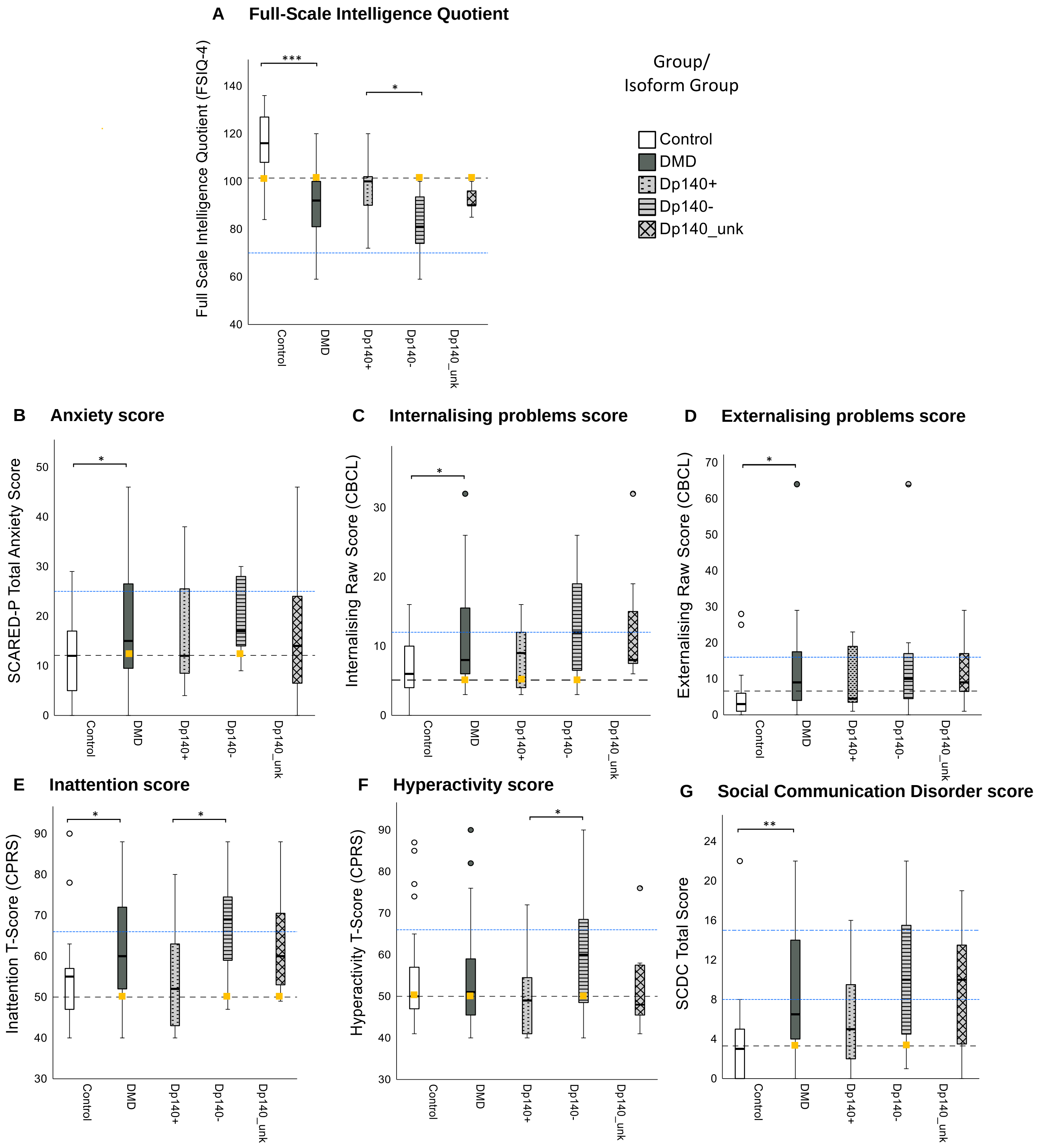

Supplementary Figure 3. Neuropsychological assessment scores in DMD vs. Control groups and DMD isoform subgroups. Box plots of neuropsychological assessment scores: solid bars in box plots indicate median values; error bars show the interquartile range calculated with inclusive median; outliers indicated with circle markers. DMD isoform subgroups shown in pattern bars: Dp140+ = Dp140 isoform expressed (n=12); Dp140- = Dp140 isoform not expressed (n=11); Dp140_unk = uncertain Dp140 expression (n=7). Black dashed line indicates normative data mean value; yellow squares indicate significant differences between group scores and normative data; blue dotted line indicates threshold for clinically significant symptoms. Between groups comparisons performed with independent samples *t*-tests, using alpha level of P=.05 (data shown in Supplementary Table 1). * P<.05; ** P<.01; *** P<.001. (**A**) Full-Scale Intelligence Quotient (FSIQ-4) assessed using Wechsler Abbreviated Intelligence Scale – 2nd Edition. Scores ≤70 indicate intellectual disability. (**B**) Anxiety scores on the Screen for Child Anxiety Related Disorders, Parent version (SCARED-P). (**C**) & (**D**) Internalising problems and Externalising problems scores assessed using parent-report Child Behavior Checklist (CBCL). (**E**) & (**F**) Inattention and Hyperactivity scores assessed using Conners' Parent Rating Scale-Revised, short version (CPRS). T-scores shown graphically to enable representation of clinically significant cut-off score (T-score ≥65), but significance testing performed on raw scores (see Supplementary Table 1). (**G**) Social communication problems assessed using parent-report Social Communication Disorders Checklist (SCDC). Scores ≥8 indicate likely neurodevelopmental disturbance (blue dotted line); scores >15 (blue dot/dash line) strongly suggestive of autism spectrum disorder

Supplementary Table 4. Correlation of neuropsychiatric measures with (A) Full-scale IQ and (B) Primary unconditioned physiological responses.

A Correlation coefficients for Full-scale IQ vs. neuropsychiatric and physiological outcomes

	**Anxiety**		**Internalising**		**Externalising**		**Social comm.**		**Inattention**		**Hyperactivity**		**SCR_{UC}[c]**		**ΔHR_{UC}[d]**	
	***rho*[a]**	***P*[b]**	***rho***	***P***	***rho***	***P***	***rho***	***P***	***rho***	***P***	***rho***	***P***	***rho***	***P***	***rho***	***P***
All (n=56)	**-0.30**	***.03***	**-0.30**	***.03***	**-0.30**	***.03***	**-0.35**	***.009***	**-0.38**	***.004***	0.069	*.62*	-0.003	*.98*	-0.02	*.87*
DMD (n=31)	-0.04	*.83*	0.07	*.71*	-0.01	*.98*	-0.14	*.46*	**-0.42**	***.02***	-0.05	*.78*	0.21	*.29*	-0.16	*.42*
Control (n=25)	-0.18	*.40*	-0.15	*.49*	0.02	*.93*	0.14	*.52*	-0.03	*.89*	0.39	*.05*	0.39	*.06*	-0.31	*.14*
DMD_Dp140+ (n=12)	0.28	*.38*	0.15	*.64*	0.26	*.42*	0.32	*.31*	-0.20	*.54*	0.34	*.28*	0.29	*.41*	-0.31	*.33*
DMD_Dp140- (n=11)	-0.16	*.65*	0.39	*.24*	-0.22	*.53*	-0.28	*.40*	-0.41	*.21*	-0.03	*.92*	0.27	*.42*	0.18	*.60*

B Correlation coefficients for unconditioned physiological responses vs. neuropsychiatric symptom scores

		**Anxiety**		**Internalising**		**Externalising**		**Social comm.**		**Inattention**		**Hyperactivity**	
**i. Adjusted for IQ**		***ρ*[e]**	***P***	***ρ***	***P***	***ρ***	***P***	***ρ***	***P***	***ρ***	***P***	***ρ***	***P***
SCR_{UC}[c]	All	**0.38**	**.01**	**0.33**	**.03**	0.13	.40	0.27	.07	0.05	.74	0.11	.45
	DMD	0.37	.08	0.27	.22	0.17	.44	0.33	.13	0.10	.65	0.19	.39
	Control	0.22	.32	0.04	.86	-0.07	.76	-0.09	.68	-0.05	.83	0.01	.96
	Dp140+	0.32	.40	0.24	.53	0.11	.78	0.12	.76	-0.14	.71	-0.02	.96
	Dp140-	**0.75**	**.01**	0.58	.08	0.34	.33	0.54	.11	0.41	.23	0.25	.48
ΔHR_{UC}[d]	All	0.02	.91	-0.05	.75	-0.15	.32	-0.13	.37	-0.02	.92	-0.001	.99
	DMD	-0.07	.77	0.03	.88	-0.20	.36	-0.02	.94	-0.02	.92	0.002	.99
	Control	0.23	.31	0.10	.65	-0.07	.75	-0.15	.51	0.008	.97	0.01	.95
	Dp140+	-0.11	.77	-0.15	.70	-0.13	.75	0.08	.84	-0.21	.59	0.19	.63
	Dp140-	0.60	.07	0.46	.18	-0.02	.95	0.12	.74	-0.29	.41	-0.43	.21
**ii. Unadjusted for IQ**		***rho***	***P***	***rho***	***P***	***rho***	***P***	***rho***	***P***	***rho***	***P***	***rho***	***P***
SCR_{UC}	All	**0.29***	**.04**	0.23	.10	0.18	.21	0.17	.25	-0.10	.95	0.14	.31
	DMD	0.28	.15	0.29	.13	0.22	.26	0.21	.29	0.00	.99	0.20	.31
	Control	0.12	.57	-0.01	.96	-0.07	.73	-0.16	.45	-0.32	.13	-0.02	.92
	Dp140+	0.30	.41	0.15	.69	-0.14	.70	0.03	.93	-0.07	.84	0.25	.49
	Dp140-	0.58	.06	**0.73**	**.01**	**0.64**	**0.03**	0.39	0.24	0.13	.71	0.11	.74
ΔHR_{UC}	All	0.10	.45	0.06	.66	-0.21	.13	-0.15	.27	-0.12	.93	-0.07	.60
	DMD	0.13	.49	0.11	.56	-0.17	.35	-0.11	.56	-0.07	.72	-0.14	.44
	Control	0.20	.34	0.22	.30	-0.09	.67	0.007	.97	0.24	.25	0.00	.99
	Dp140+	-0.02	.95	0.13	.69	-0.16	.61	-0.17	.60	-0.03	.92	0.07	.82
	Dp140-	0.47	.14	0.30	.37	0.21	.53	0.02	.95	-0.34	.31	-0.43	.18

[a] *rho* = Spearman correlation coefficient.
[b] *P* = signficance, with significance level alpha of *P*=.05. P-values <.05 and corresponding correlation statistics are shown in bold.
[c] SCR_{UC} = Unconditioned skin conductance response to the first 'threat' trial with conditioned stimulus, CS+, in microSiemens (μS).
[d] ΔHR_{UC} = Unconditioned change in heart rate response to the first CS+ 'threat' trial, in beats per minute (bpm).
[e] *ρ* = Partial correlation coefficient, controlling for full-scale IQ.

Supplementary Figure 4

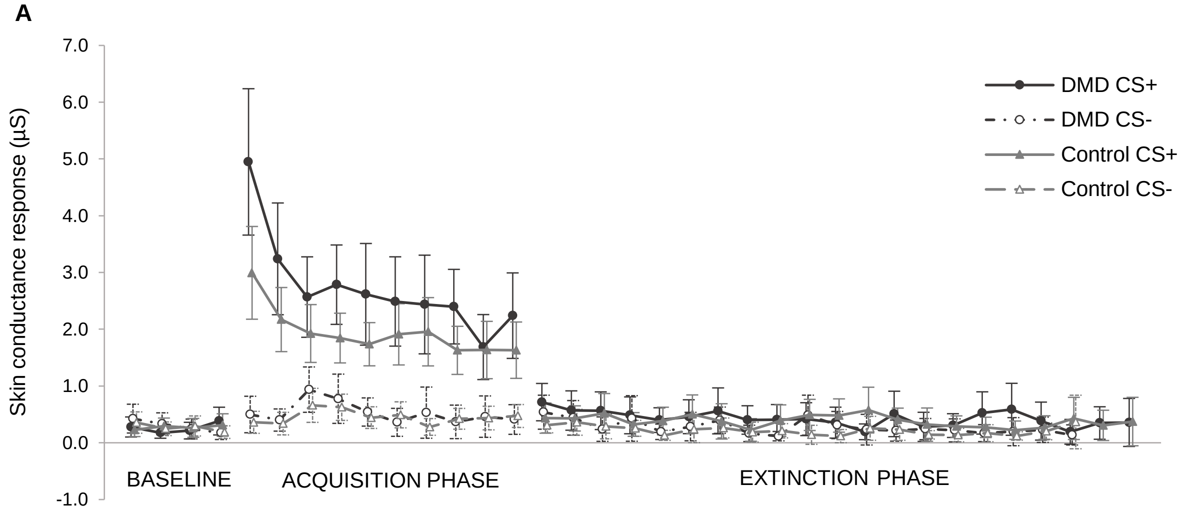

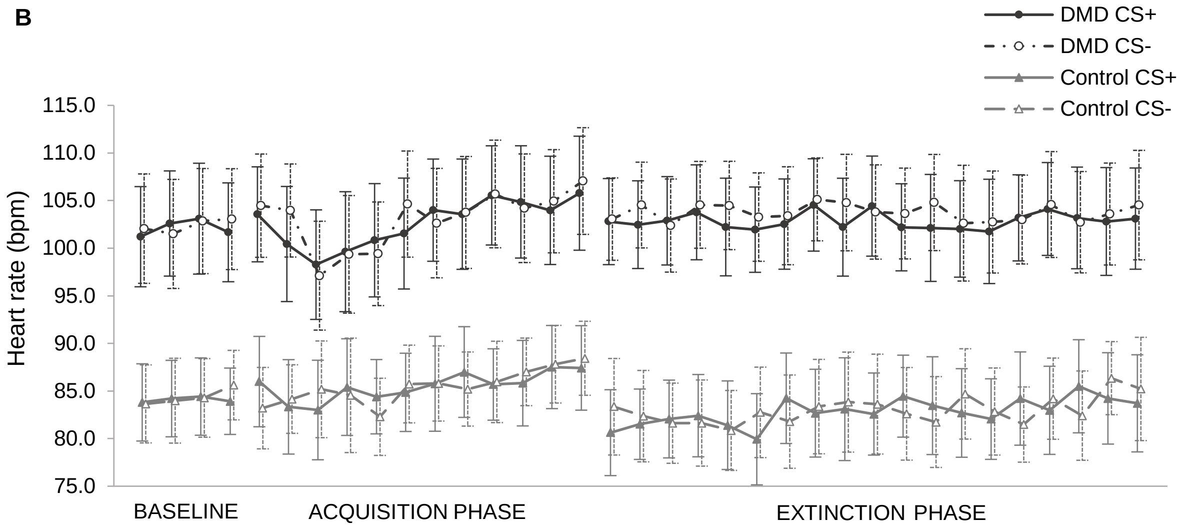

Supplementary Figure 4: Skin conductance response and heart rate data for each CS+ and CS- trial in all phases of the task.

(**A**) Skin conductance responses (SCR, measured in microSiemens, µS) for DMD and Control groups in CS+ and CS- trials. (**B**) Absolute heart rate (HR, measured in beats per minute, bpm). Baseline is the initial familiarisation phase where both CS+ and CS- are presented for four trials each but without the aversive unconditioned stimulus. Acquisition phase comprises 24 trials (12 of each CS+ and CS-), and Extinction phase comprises 40 trials. Error bars in show 95% Confidence Intervals for each data point.

Supplementary Table 5. Primary outcome measures without adjustment for IQ.

Primary SCR and HR metrics (not adjusted for IQ)				
**Group**	**DMD**	**Control**	**DMD Dp140+**	**DMD Dp140-**
Mean SCR_{UC}[a] in µS *(se; 95% CI)*[b]	4.9 *(0.6; 3.8, 6.1)*	3.0 *(0.6; 1.8, 4.2)*	5.4 *(1.1; 3.0, 7.8)*	5.0 *(2.7; 7.2)*
Mean ΔHR_{UC}[c] in bpm *(se; 95% CI)*	-6.4 *(2.0; -10.5, -2.4)*	-0.8 *(2.2; -5.3, 3.7)*	-7.3 *(2.8; -13.1,-1.4)*	-2.6 *(2.9; -8.7, 3.5)*
SCR_{EXT}[d] in µS *(se; 95% CI)*	0.7 *(0.2; 0.4, 1.0)*	0.4 *(0.2; 0.1, 0.8)*	0.6 *(0.3; 0.04, 1.2)*	1.2 *(0.3; 0.6, 1.8)*

Between group comparisons (not adjusted for IQ)												
**Group**	**DMD vs. Control**			**DMD Dp140+ vs. Control**			**DMD Dp140- vs. Control**			**DMD Dp140- vs. Dp140+**		
**Outcome measure**	**Mean diff. *(95% CI)***	η^2	**Sig., *P*[f]**	**Mean diff. *(95% CI)***	η^2	**Sig., *P***	**Mean diff. *(95% CI)***	η^2	**Sig., *P***	**Mean diff. *(95% CI)***	η^2	**Sig., *P***
SCR_{UC} (in µS)	2.0 *(0.3, 3.6)*	0.11	**.02**	2.4 *(0.4, 4.3)*	0.16	**.02**	2.0 *(0.03, 3.9)*	0.11	**.047**	-0.4 *(-3.7, 2.8)*	0.004	.79
ΔHR_{UC} (in bpm)	-5.6 *(-11.6, 0.4)*	0.06	.07	-6.5 *(-14.8, 1.9)*	0.07	.13	-1.8 *(-10.6, 7.0)*	0.01	0.68	4.7 *(-3.8, 13.1)*	0.06	.26
SCR_{EXT} (in µS)	0.28 *(-0.16,0.72)*	0.04	.20	0.17 *(-0.31, 0.65)*	0.02	0.47	0.78 *(0.14, 1.4)*	0.17	**0.02**	0.6 *(-0.2, 1.4)*	0.14	.14

[a] SCR_{UC} = Unconditioned skin conductance response to the first 'threat' trial with conditioned stimulus, CS+, in microSiemens (µS).
[b] se = standard error; CI = confidence interval.
[c] ΔHR_{UC} = Unconditioned change in heart rate response to the first CS+ 'threat' trial, in beats per minute (bpm).
[d] SCR_{EXT} = Conditioned skin conductance response to the first CS+ trial of the Extinction phase, in µS.
[e] η^2 = effect size of Group (Eta squared)
[f] Significance (Sig.) testing using univariate analysis of variance between groups/subgroups, taking alpha of *P*=.05; *P*-values <.05 are highlighted in bold. *F* = effect size of Group/Isoform group. Bonferroni adjustment made for multiple comparisons.

Supplementary Table 6. SCR discrimination between CS+ and CS- stimuli within groups (A); Differential SCR (SCR_{Diff}) within groups in the First Interval Response window (B) showing conditioned response acquisition and Second Interval Response window (C) showing habituation.

A Mean differences between SCR_{CS+} and SCR_{CS-} in µS by block within groups[a]

		DMD		Control	
**Block**		**Mean difference SCR_{CS+} vs. SCR_{CS-} in µS *(95% CI)***	**Sig., *P***[b]	**Mean difference SCR_{CS+} vs. SCR_{CS-} in µS *(95% CI)***	**Sig., *P***
Familiarisation block		0.006 *(-0.23, 0.25)*	.96	0.02 *(-0.23, 0.27)*	.86
Acquisition blocks	ACQ1	**2.80 *(2.57, 3.04)***	**<.001**	**1.7 *(1.5, 2.0)***	**<.001**
	ACQ2	**2.01 *(1.79, 2.33)***	**<.001**	**1.4 *(1.1, 1.6)***	**<.001**
	ACQ3	**1.74 *(1.46, 2.01)***	**<.001**	**1.1 *(0.8, 1.4)***	**<.001**
Extinction blocks	EXT1	0.18 *(-0.10, 0.43)*	.21	0.09 *(-0.18, 0.35)*	0.52
	EXT2	0.16 *(-0.12, 0.44)*	.27	0.14 *(-0.14, 0.41)*	0.33
	EXT3	0.07 *(-0.23, 0.36)*	.65	**0.37 *(0.10, 0.64)***	**0.007**
	EXT4	0.23 *(-0.07, 0.54)*	.13	0.13 *(-0.17, 0.42)*	0.40
	EXT5	0.21 *(-0.11, 0.53)*	.20	0.15 *(-0.14, 0.43)*	0.32

B Conditioned response acquisition: Between groups comparison of SCR_{Diff}[c] **in First Interval Response window of Acquisition phase**[d]

**Block**		**Mean difference DMD vs. Control in µS *(95% CI)***	**Sig., *P***
Acquisition blocks	ACQ1	-0.14 *(-0.71,0.42)*	.62
	ACQ2	0.17 *(-0.29,0.63)*	.45
	ACQ3	-0.21 *(-0.66,0.24)*	.35

C Conditioned response habituation: SCR_{CS+} in Second Interval Response window of Acquisition phase[e]

	DMD		Control	
**Block comparison**	**Mean difference in SCR_{CS+} in µS *(95% CI)***	**Sig., *P***	**Mean difference in SCR_{CS+} in µS *(95% CI)***	**Sig., *P***
ACQ1 vs. ACQ2	**-0.86 *(-1.3, -0.44)***	**<.001**	-0.42 *(-0.45, 0.01)*	.07
ACQ1 vs. ACQ3	**-1.2 *(-1.7,-0.80)***	**<.001**	**-0.65 *(-1.1,-0.23)***	**<.001**

[a] SCR_{CS+} is the skin conductance response in CS+ 'threat' trials; SCR_{CS-} is the skin conductance response in CS- 'safe' trials. The difference between these two values indicates the degree of discrimination between the two trial types, showing the mean difference for each block (eight trials; four of each type) in both DMD and Control groups.

[b] All statistical analyses were conducted using linear mixed models analysis. 95% CI = 95% confidence interval; Sig. = significance level alpha, *P*=.05. *P*-values <.05 shown in bold.

[c] SCR_{Diff} is the differential SCR between SCR_{CS+} and SCR_{CS-} in contiguous trials.

[d] First Interval Response (FIR) window captured responses occurring 0-6s after CS onset, before the 'threat' stimulus was presented, allowing responses to the CS alone to be identified. Responses in this window indicate the degree of conditioned response learning.

[e] Second Interval Response window captured responses occurring 6-12s after CS onset and captured the response to the unconditioned 'threat' stimulus. Responses in this window indicate the degree of habituation to the unconditioned stimulus.
